# Swashing: a propulsion-independent form of bacterial surface migration

**DOI:** 10.1128/jb.00323-25

**Published:** 2025-11-03

**Authors:** Justin Panich, Eric M. Dudebout, David F. Blair, Navish Wadhwa

**Affiliations:** 1 School of Biological Sciences, University of Utah, Salt Lake City, Utah, USA; 2 Biological Systems and Engineering Division, Lawrence Berkeley National Laboratory, Berkeley, California, USA; 3 Biodesign Center for Mechanisms of Evolution, Arizona State University 7864, Tempe, Arizona, USA; 4 Center for Biological Physics, Arizona State University 7864, Tempe, Arizona, USA; 5 Department of Physics, Arizona State University 7864, Tempe, Arizona, USA; Geisel School of Medicine at Dartmouth, Hanover, New Hampshire, USA

**Keywords:** cell motility, surface migration, fermentation, fluid flow, osmotic gradient, flagella, range expansion

## Abstract

Physical forces play a crucial role in microbial behavior. In free-swimming bacteria, the hydrodynamics of flagellar propulsion govern motility. On surfaces, physical interactions are equally important though often complex and easily overlooked. Surface migration occurs in many flagellated bacteria and has been linked to diverse mechanisms, such as flagellar propulsion, surface tension, osmolyte production, or combinations thereof. However, the relative importance of these physical effects remains unclear. Here, we describe “swashing,” a form of surface movement in which bacteria migrate without active propulsion. Mutants of *Salmonella enterica* and *Escherichia coli* lacking flagellar filaments and motility proteins exhibit surface migration comparable to wild-type strains. Swashing is supported by fermentable sugars and inhibited by surfactants. We propose a model in which fermentation at the colony edge creates an osmotic gradient that draws water from the agar, generating fluid flows that propel the colony outward. Overall, our findings show that flagellar propulsion is dispensable for surface migration; instead, fermentation-driven fluid flows may be sufficient to enable bacterial spreading.

**IMPORTANCE:**

Bacteria move on surfaces using a variety of mechanisms, with important implications for their growth and survival in both the clinical setting (such as on the surface of medical devices) and the wild. Surface motility in Gram negative model species *S. enterica* and *E. coli* has been extensively studied and is believed to depend on flagellar propulsion. Here, we show surface expansion in these species even in the absence of propulsion by the flagella. We suggest that this flagella-independent movement is tied to fermentation and resulting osmotic flow: As cells ferment sugars, they create local osmolarity gradients, which generate a wave of fluid that drives expansion.

## INTRODUCTION

**Fig 1:**
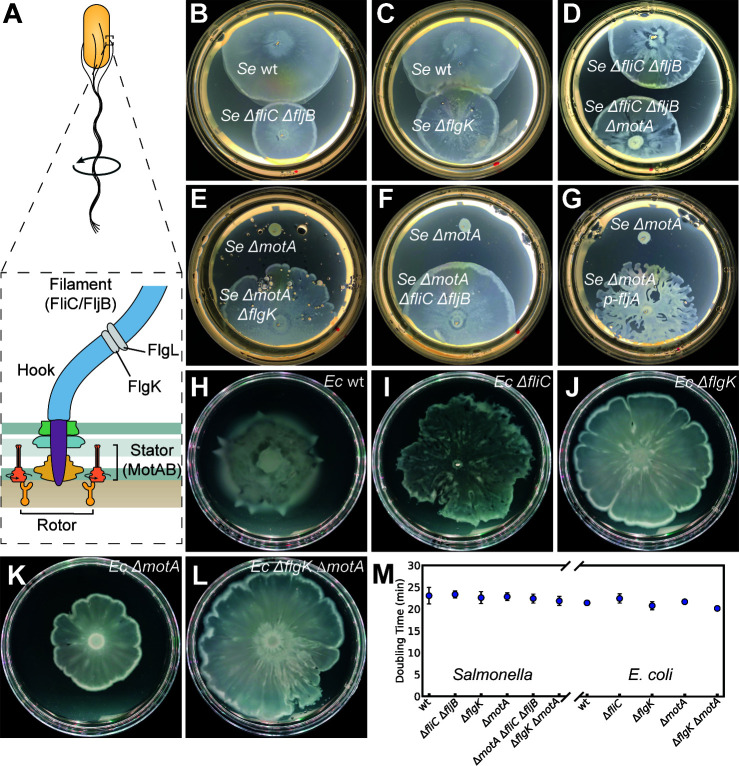
Propulsion-independent surface migration in *S. enterica* LT2 and *E. coli*. (**A**) Schematic of the bacterial flagellar motor. The stator complex (MotAB) powers rotation of the rotor, which connects to the filament (FliC/FljB in *Salmonella*; FliC in *E. coli*) via the hook and junction proteins FlgK and FlgL. (**B–D**) Migration of both wild-type and filamentless *Salmonella* strains on soft agar plates. (**E–G**) *Salmonella* ∆*motA* mutants, which produce non-rotating filaments, fail to migrate. Migration is restored by disrupting filament assembly through deletion of *flgK* (hook-filament junction), *fliC*/*fljB* (flagellins), or by FljA overexpression, which represses flagellin translation. Plates in (**B and C**) were incubated for 10 h to avoid overgrowth of wild-type *Salmonella*.; (**D–G**) for 12 h. (**H–J**) Surface migration of wild-type and filamentless *E. coli* strains lacking either flagellin (FliC) or the junction protein (FlgK). (**K and L**) In *E. coli*, a ∆*motA* strain shows impaired migration. Like in *Salmonella*, migration is rescued by further removal of the flagellar filament by disrupting filament assembly through deletion of *flgK*. Plates in (**H**) were incubated for 12 h to avoid overgrowth of wild-type *E. coli*.; (**I–L**) for 16 h. (**M**) Doubling times of *Salmonella* and *E. coli* strains grown in LB +0.5% glucose at 37°C from 1:100 dilutions of overnight cultures.

The ability to move allows many species of bacteria to seek and colonize favorable environments, avoid unfavorable conditions, and form complex macroscopic structures such as biofilms or fruiting bodies (1–4). Mechanisms of bacterial motility have been intensively studied and can be grouped into different classes based on underlying mechanisms and the setting (e.g., liquid vs solid media) where the behavior occurs (1, 5). Many species swim in liquid media using flagella (Fig. 1A), which harness the membrane ion gradient to drive rotation of helical filaments that function as propellers (6–9).

Movement on surfaces occurs by diverse mechanisms, including the extension and retraction of specialized pili (twitching) (10), the movement of membrane-bound adhesins along the cell surface (gliding) (11), by surfactant-assisted but largely passive growth-based mechanisms (sliding) (12), or by collective motion of a large number of cells within a thin layer of fluid on the surface (swarming) (13–15). Swarming on agar surfaces has been observed in diverse genera, including *Proteus* (16), *Vibrio* (17), *Bacillus* (18), and *Clostridium* (19), as well as in the Gram-negative model species *Escherichia coli* and *Salmonella typhimurium* (15).

While swarming has been studied extensively (20–30), its mechanism is not completely understood. The physical factors that can drive colony expansion may include flagellar propulsion, surfactant-driven Marangoni flows, and osmotically driven fluid fluxes in and out of the agar (15, 31–35). Flagellar rotation generates thrust that can push on the colony boundary and thereby cause outward expansion (6, 15, 33). Marangoni flows derive from gradients of surface tension that apply a force at the air-water interface, pulling the fluid and generating a flow (36). Many bacteria, like *Pseudomonas aeruginosa*, exploit Marangoni flows by producing surfactants that generate a surface tension gradient, pulling the colony outwards (33, 35). Osmotic-driven fluid fluxes involve osmotic pressure generated within a colony (31). Bacteria generate osmolytes that draw water from the agar into the colony, increasing the fluid volume that the colony occupies and driving volumetric expansion (32, 33, 37). While all these physical factors can play a role in colony expansion, their relative importance for bacterial surface migration is unclear.

Here, we describe “swashing,” a form of surface migration in *Salmonella* and *E. coli* that does not require active propulsion. Mutants of *S. enterica* and *E. coli* lacking flagellar filaments and motility proteins move on agar plates at more than half the rate of wild-type strains. Swashing relies on fermentable sugars and is inhibited by surfactants, distinguishing it from other mechanisms of surface migration. We propose a model in which fermentation at the colony edge creates osmotic gradients that drive water flow out of the agar, pushing cells outward. Swashing offers a new mode of bacterial range expansion independent of flagellar propulsion and clarifies the role of physical factors involved in bacterial surface migration.

## MATERIALS AND METHODS

### Bacterial strains and cultures

The *Salmonella* and *E. coli* strains used in this study are listed in Tables S2 and S3, respectively. *E. coli* and *Salmonella* strains were grown in LB liquid media at 37°C with appropriate antibiotics. When plating on solid media, LB media was solidified with 1.5% (wt/vol) agar, supplemented with appropriate antibiotics, at concentrations of 10 µg/mL for tetracycline, 100 µg/mL for ampicillin, and 50 µg/mL for spectinomycin.

### Strains and vector construction

Karlinsey’s Lambda red recombineering method (38) was used to create the knockout strains of *E. coli* and *Salmonella* used here. Briefly, DNA oligomers with homology to chromosomal targets and antibiotic resistance cassettes were amplified by PCR from the pKD46 plasmid. The amplified DNA was purified by ethanol precipitation and was then electroporated into the desired strain/species. Cells were allowed to recover in liquid LB at 37°C for 1 h and were then plated on selective media. For clean deletions, a selection and counter-selection method of a tetracycline cassette (selection) with tetracycline-sensitive plates (counter-selection) was used. In these cases, 80 bp ssDNA oligomers were used for the deletion of the tetracycline cassette in the targeted region without PCR amplification. All chromosomal mutations were made with the first and last five intact codons to mitigate potential gene expression polarity artifacts.

In addition to the Karlinsey’s method, transduction methods were also used for strain construction in *Salmonella*. For transduction methods, phage P22 was used (39).

Plasmids used are summarized in Table S4. All plasmids were constructed using the Gibson Assembly protocols (40) and were transformed into *E. coli* and *Salmonella* cells using standard transformation protocols.

### Surface migration assays

*Salmonella* migration plates were prepared with Luria-Bertani broth (LB; 1.0% tryptone, 0.5% NaCl, 0.5% yeast extract), 0.55% Difco agar, and 0.5% glucose (or other sugars as specified). After pouring 20 mL of the media into Petri dishes, plates were left to dry and solidify at room temperature overnight and then incubated at 32°C in a humidified chamber for 24 h before inoculation. A 4 µL aliquot of saturated *Salmonella* culture (grown in liquid LB) was spotted onto the surface. Plates were incubated at 37°C in approximately 100% humidity for 12 h or as otherwise indicated.

*E. coli* plates were prepared similarly, using LB with 0.5% Eiken agar (Eiken Chem Co., Japan) and 0.5% glucose. After pouring 25 mL of media, plates were dried for 8 h at room temperature. A 2 µL aliquot of saturated *E. coli* culture was then inoculated and left to dry for 5 min in a laminar flow hood until the drop evaporated. Plates were incubated at 37°C in 90%–100% humidity for 16 h or as specified.

Because wild-type *Salmonella* and *E. coli* migrated slightly faster than filamentless strains, their incubation times were shortened to prevent overgrowth of the agar surface.

### Growth comparisons

Wells of a Greiner clear-bottom 96-well microplate were filled with 198 µL of LB + 0.5% glucose and inoculated with 2 µL of saturated culture (1:100), grown in LB media. A lid was placed over the microplate to prevent evaporation, and the microplate was subsequently placed in a BioTek H1 plate reader. The plate reader was set to shake using the orbital shake function at 37°C and recorded OD600 at half-hour intervals for 10 h. A sigmoidal function was fit to the growth curve data in Python. The doubling time was then extracted from the exponential growth phase of the data. For each strain and condition tested, doubling times were obtained from two to four biological replicates, with two technical replicates per biological replicate.

### Preparation of bromothymol blue solutions and media acidification assays

To examine acidification of the media, we added the pH indicator, bromothymol blue, to the motility assay plates. Bromothymol blue is green at neutral pH, yellow at pH <6.0, and blue at pH >7.6. Bromothymol blue stock solution was prepared by adding 100 mg bromothymol blue to 10 mL of 4% NaOH (vol/vol), to which 10 mL of a 95% (vol/vol) Ethanol solution was then added. The resulting solution was then further diluted with ddH2O to a final volume of 100 mL, yielding a final concentration of 1 mg bromothymol blue/mL. 1.25% (vol/vol) bromothymol blue solution was added to the media to yield a final concentration in the plates of 12.5 µg/mL.

To quantify the pH of swashing *Salmonella*, a glass pH electrode was used to measure the H^+^ ion activity at various locations in the colony.

### Microscopy and cell tracking

*E. coli* and *Salmonella* strains were grown in liquid cultures to mid-log phase, OD600 of 0.4 to 0.6. Cells were pelleted at 1,500 *g* for 5 min and were resuspended in motility buffer (10 mM potassium phosphate, 0.10 mM EDTA, pH 7.5) to a final OD of approximately 0.02. 80 µL of diluted cell culture was pipetted into a tunnel slide (formed by a cover slip fixed on a glass slide with two pieces of double-sided sticky tape). Suspended cells were imaged at 25 fps on a Nikon Eclipse Si phase contrast microscope fitted with a BFS-U3-28S5M-C USB 3.1 Blackfly S, Monochrome Camera. Cell motility videos were then analyzed in ImageJ using Trackmate (41).

### Measurements of acetate, formate, and glucose concentrations within agar

Plates were sampled by manually extracting 10 or more agar plugs from each plate into a Pasteur pipette, at various positions relative to the margin of an expanding colony. Plugs were combined in a microfuge tube and spun to pellet the agar, and then concentrations of acetate, formate, or glucose in the supernatant were measured using enzyme-based assays (Abcam assay kits ab204719, ab111748, and ab65333, respectively).

### Optical profilometry measurements

An *E. coli* ∆*flgK* swashing colony was allowed to grow for 10 h. The procedure for the surface migration was the same as described above, except the volume of media poured into the Petri dish was increased to 45 mL to ensure the level of the agar was near the top of the Petri dish. The swashing colony was brought to a ZeScope Optical Profilometer, where the colony edge topology was recorded. Output from the profilometer was analyzed and plotted using MATLAB scripts.

### Swashing colony imaging and analysis

Imaging of the *E. coli* surface motility assays was performed in a modified, custom-built imaging incubator. An incubator was fitted to have a Canon EOS Rebel SL3 Digital SLR Camera with EF-S 18–55 mm lens, centered above the plate. Images were taken in end-point single-shot fashion. The imaging apparatus was fit to ensure minimal loss of humidity or temperature. The incubator was lit using high-density white light Adafruit LED strips, parallel and slightly above the plane of the plates. Multiple layers of diffuser paper were placed over the LED strips to ensure optimal lighting conditions and prevent reflections.

Imaging of the *Salmonella* surface motility assays was performed on a black background with lighting parallel and level with the plane of the plates.

Image processing and analysis were performed in Python.

## RESULTS

### Propulsion-independent surface movement in *Salmonella*

We discovered swashing in an experiment first intended as a negative control. In this experiment, we examined the migration of a *Salmonella* strain, which had both flagellin genes *fliC* and *fljB* deleted, on soft agar plates (LB, 0.5% glucose, 0.55% agar). The ∆*fliC*∆*fljB* mutant assembles the hook-basal body (HBB) but lacks the flagellar filament required for propulsion (Fig. 1A). Surprisingly, the filamentless ∆*fliC*∆*fljB* cells migrated on soft agar plates albeit at a rate slower than motile wild-type cells (Fig. 1B). Cells grown in liquid culture were non-swimming when examined under the microscope (Fig. S1) and, as expected, failed to migrate inside the softer (0.3% agar) “swim” plates (Fig. S2), where they require flagellar propulsion to move.

Prompted by this finding, we examined other non-swimming mutants. A ∆*flgK* mutant, which lacks the hook-filament junction and assembles only the HBB, also migrated rapidly on soft agar plates (Fig. 1C). Like the ∆*fliC*∆*fljB* mutant, the ∆*flgK* mutant was immotile when examined under the microscope (Fig. S1) and failed to migrate in 0.3%-agar motility plates (Fig. S2). A ∆*flgL* mutant, which also only assembles the HBB, displayed the same phenotype as ∆*flgK* (Fig. S3). Thus, mutants that assemble just the HBB, though immotile in liquid and in swim plates, migrate rapidly on the surface of soft agar plates. The hook structure still present in these mutants might, in principle, enable some propulsion. To test this possibility, we prevented hook rotation by deleting the motility gene *motA* (9), in the filamentless ∆*fliC*∆*fljB* background. Migration was not impaired by paralysis of the hook (Fig. 1D), and differences in expansion rates of these various strains were not correlated to any differences in their growth rates (Fig. 1M). Failure to polymerize the filament in the HBB-assembling strains may enhance FlgM export and thus FliA activity (42). While a ∆*fliA*∆*flgK* mutant did not swash (Table S1), a ∆*flgM*∆*flgK* mutant (expected to have elevated FliA activity) also failed to swash (Table S1), suggesting that increased FliA activity alone does not explain migration.

The rapid migration of the ∆*fliC*∆*fljB*∆*motA* strain, which lacks flagellar filaments, contrasts with the non-migratory phenotype of the ∆*motA* mutant reported by Harshey and Matsuyama (15) and reproduced here (Fig. 1E). As shown in Fig. 1E through G, migration of the ∆*motA* mutant is restored when filament assembly is disrupted—by deletion of the flagellin genes, deletion of *flgK*, or overexpression of the regulatory protein FljA, which inhibits *fliC* translation. Migration is also restored when ∆*motA* cells are mixed with robustly swashing ∆*flgK* cells (Fig. S4). These results suggest that the migration defect in ∆*motA* arises from the presence of paralyzed filaments, not the absence of active propulsion, and can be alleviated by reducing filament-induced hindrance (via de-densification of cells with paralyzed filaments).

### Propulsion-independent surface movement in *E. coli*

To examine the generality of swashing, we repeated the experiments described above for *Salmonella* with *E. coli* K12 (strain MG1665). With nutrient sources like those used for *Salmonella* (LB supplemented with 0.5% glucose), w.t. *E. coli* swarmed reproducibly at rates similar to w.t. *Salmonella* (Fig. 1H). As in *Salmonella*, *E. coli* mutants lacking the flagellar filament, whether due to loss of flagellin FliC or the hook-filament connector FlgK, retained the ability to move at a significant rate on soft agar plates (Fig. 1I and J). Migration of *E. coli* mutants that lacked the motility protein MotA decreased, but did not entirely cease (Fig. 1K). As in *Salmonella*, rapid migration in the ∆*motA* mutant was rescued by further deletion of *flgK* (Fig. 1L). All mutant strains grown in liquid culture were non-swimming when examined by light microscopy (Fig. S1), did not migrate in soft-agar (0.30%) swim plates (Fig. S2), and did not demonstrate significant differences in growth rates (Fig. 1M). Thus, like S. *enterica*, *E. coli* migrates on soft agar plates in a process that does not require propulsion by the flagella.

### Surfactants inhibit swashing

Surface tension forces can promote bacterial colony expansion by generating Marangoni flows (35). This mechanism can even enable migration in non-motile bacteria, a process known as sliding (12). In documented cases—such as in *Pseudomonas*—sliding depends on bacteria-produced surfactants or other cell-surface molecules (43, 44).

**Fig 2:**
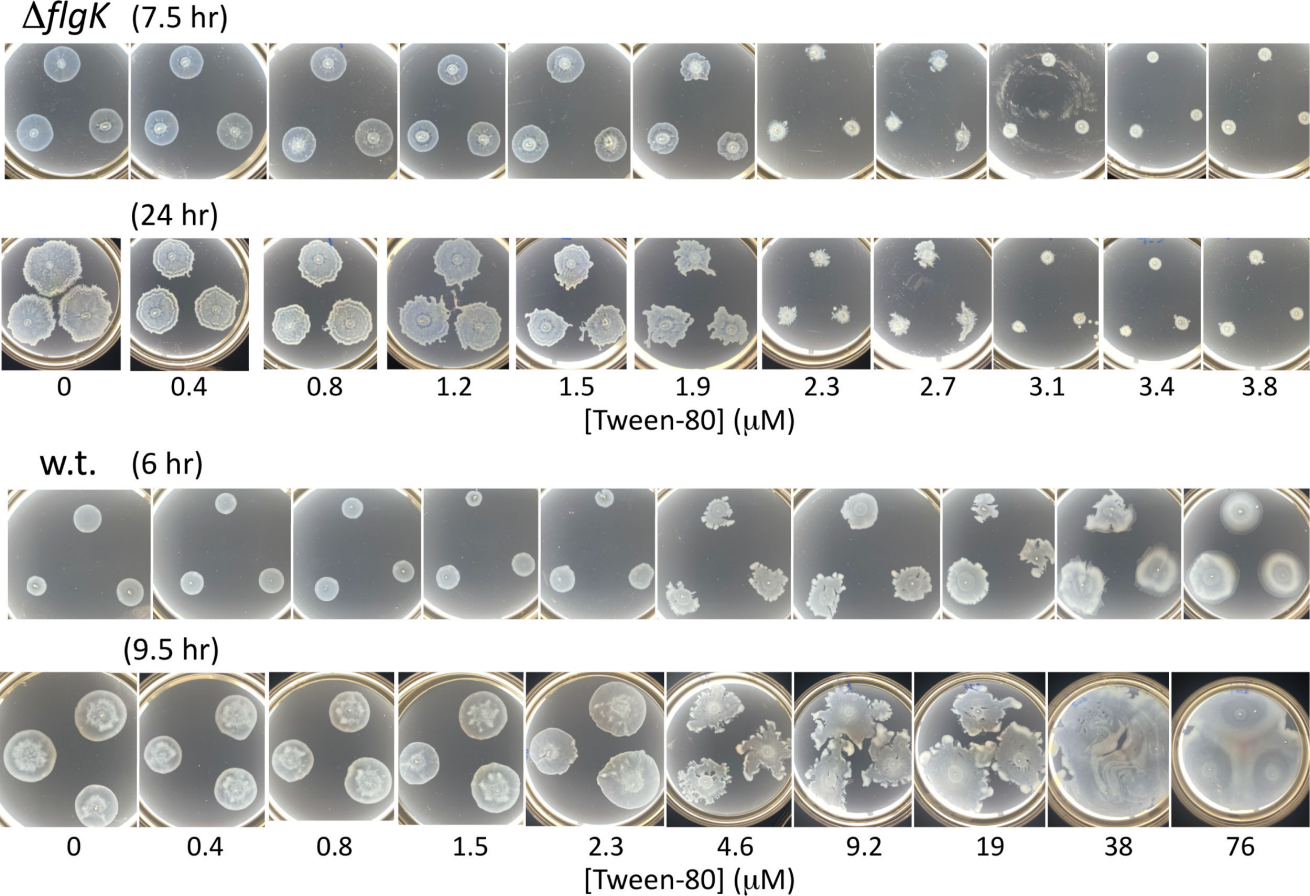
Inhibition of *Salmonella* ∆*flgK* swashing and enhancement of wild-type swarming by surfactant. Tween-80 was added to the concentrations indicated, during pouring. Plates were spotted with 4 µL of overnight culture and incubated at 37°C for the indicated times, which best captured the surfactant response in each strain.

Swashing, by contrast, does not involve surfactants. In both *Salmonella* and *E. coli*, swashing was inhibited—not aided—by the addition of the surfactant Tween-80 (Fig. 2; Fig. S5). In wild-type strains, however, swarming was enhanced by Tween-80 (Fig. 2; Fig. S5), in agreement with previous work (34, 45–48).

Tween-80 had little effect on the swashing of *Salmonella* ∆*flgK* at concentrations below 1.5 µM but inhibited it at 3 µM and above (Fig. 2; Fig. S5). Partial inhibition was observed at intermediate levels (∼2.5 µM). In contrast, Tween-80 did not inhibit wild-type swarming at any concentration tested (Fig. 2). At intermediate concentrations (∼10 µM), colonies became irregular in shape, and higher concentrations caused faster expansion. Similar trends were observed in *E. coli* (Fig. S5). Tween-80 did not impair growth at any tested concentration, indicating that swashing inhibition was not due to growth defects (Fig. S6).

### Swashing is associated with fermentation

Although the reason has been unclear, glucose is required for swarming in *Salmonella* and *E. coli* and is typically included in plates at a concentration of 0.5% (15, 49). In line with earlier findings (15), we observed that swarming of wild-type cells stopped below 0.25% glucose in *Salmonella* and 0.3% in *E. coli*. Swashing of the ∆*flgK* strain in both species showed a similar glucose dependence (Fig. S7).

**Fig 3:**
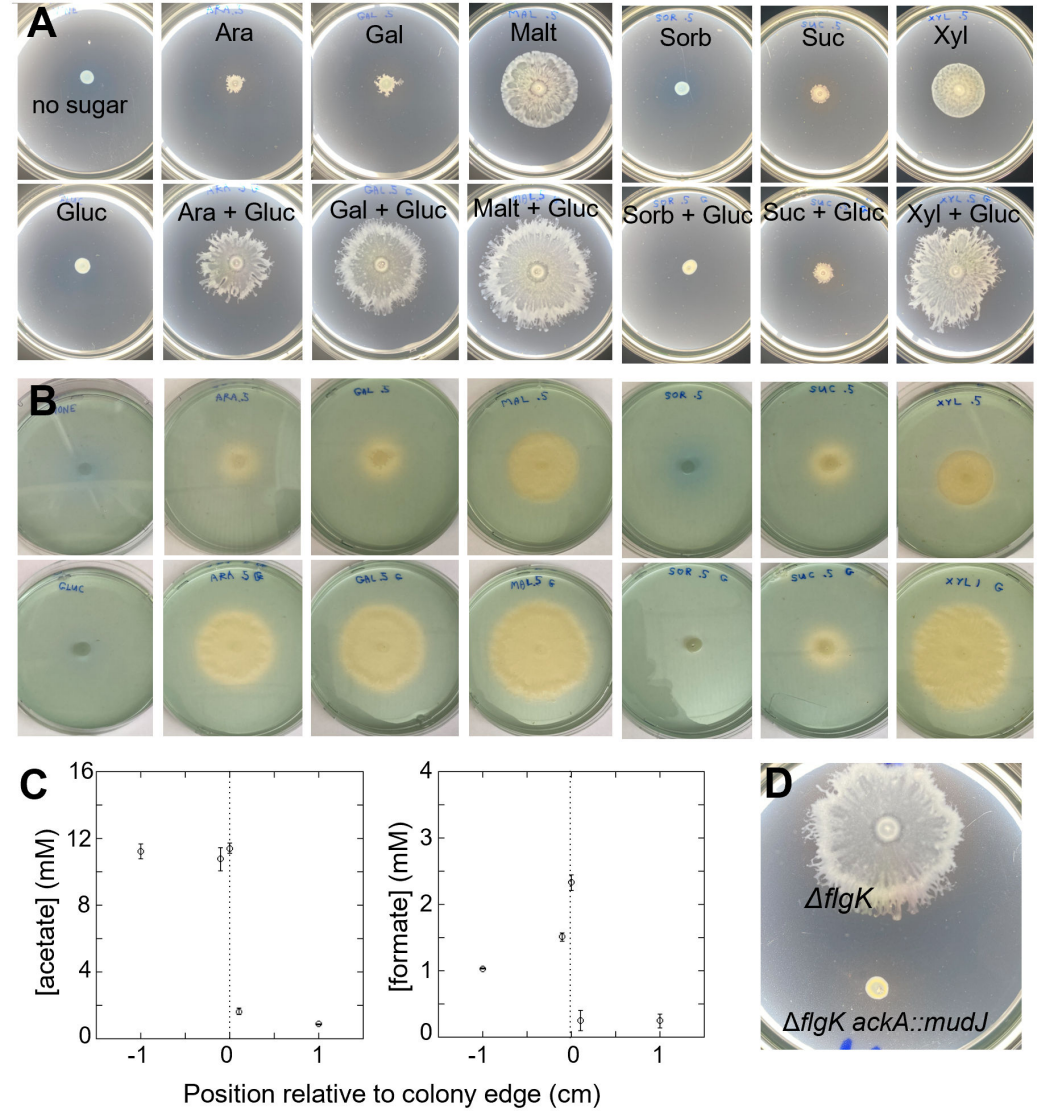
Association of swashing with fermentation. (**A**) Effects of various sugars on migration of the *Salmonella* ∆*flgK* strain and (**B**) acidification of the plates by the advancing cells. The ∆*flgK* strain was spotted on swarm plates containing the indicated sugar sources and the pH indicator bromothymol blue (1.25% (vol/vol) of 1 mg/mL). Where indicated, glucose was added to a concentration of 0.2%; other sugars were present at the standard concentration of 0.5%. Plates were spotted with 4 µL of overnight culture and incubated at 37°C for 16.5 h. In (**B**), the same plates were imaged with a white background to highlight the color change due to acidification. (**C**) Production of acetate and formate by the advancing cells. The data points are averages of six replicates, and the error bars represent the standard error for each measurement. (**D**) Effect of deleting *ackA* (encoding acetate kinase) on migration of the ∆*flgK* strain. The plate was incubated at 37°C for 17 h.

This led us to test whether other sugars could support swashing. At 0.5%, maltose and xylose supported robust swashing in *Salmonella* ∆*flgK* (Fig. 3A), while arabinose and galactose, and to a smaller extent sucrose, produced small protrusions. Adding 0.2% glucose—insufficient to support swashing on its own—enhanced swashing on maltose, xylose, arabinose, or galactose plates (Fig. 3A). Sorbose did not support swashing, with or without supplemental glucose.

The sugars that support swashing are all fermentable by *Salmonella*. At the high cell densities occurring on plates, some fermentation is expected due to limited oxygen availability, and the resulting acidification of the medium serves as a marker of this activity. Indeed, we observed acidification in plates containing the indicator bromothymol blue (1.25% (vol/vol) of 1 mg/mL) for any of the fermentable sugars (Fig. 3B). Electrode measurements of expanding *Salmonella* ∆*flgK* on 0.5% glucose confirm the acidification seen with bromothymol blue (Fig. S8). The pH of a swashing colony rapidly decreases at the margin, from 6.86 outside to 6.10 at the colony edge and 5.35 in the interior. The mixed-acid fermentation carried out by *Salmonella* produces acetate and formate as major products (50, 51). Both products were seen in the wake of migrating cells (Fig. 3C). While the glucose in the plate was only partially utilized by cells at the colony margin, glucose concentration decreased across the margin by about half the increase in acetate (Fig. S9), as expected if fermentation is the major pathway for glucose utilization. Acetate concentration was elevated and fairly uniform behind the advancing front, whereas formate spiked near the colony margin, consistent with the reuptake and subsequent oxidation of formate that has been documented in *E. coli* and *Salmonella* (52, 53). Disruption of the gene for acetate kinase, required for mixed-acid fermentation, prevented migration of the ∆*flgK* strain (Fig. 3D). Exogenous acetate added to the inoculation spot of a ∆*flgK* ∆*ackA* colony did not restore movement (Fig. S10). Together, these results suggest that fermentation—and the production of by-products like acetate and formate—plays an important role in swashing and that swashing requires not just the presence of these by-products, but dynamic changes in their concentration, driven by production at the colony front.

pH differences within the swashing colony may help direct movement. Cells behind the front experience lower pH and elevated acetate, which could suppress fermentation and break the symmetry between the inner and outer edges of the front. Consistent with this idea, spotting 1 M acetic or hydrochloric acid onto agar plates with expanding *Salmonella* ∆*flgK* colonies sharply repelled expansion, leaving persistent growth gaps. In contrast, spotting with 1 M sodium hydroxide did not inhibit swashing (Fig. S11). We next tested the effect of buffering by adding MOPS or sodium acetate to the agar before solidification. At pH 7.0, buffering with either MOPS or acetate did not block swashing, though acetate—a fermentation product as well as a buffer—slightly slowed expansion and produced patchier colonies (Fig. S12). In contrast, buffering at higher alkalinity (pH 8.0), which slows acidification, strongly suppressed swashing (Fig. S12). Together, these results suggest that pH gradients across swashing colonies shape expansion, and that swashing fails when acidification is prevented by buffering at high pH.

### Topography of a swashing colony

**Fig 4:**
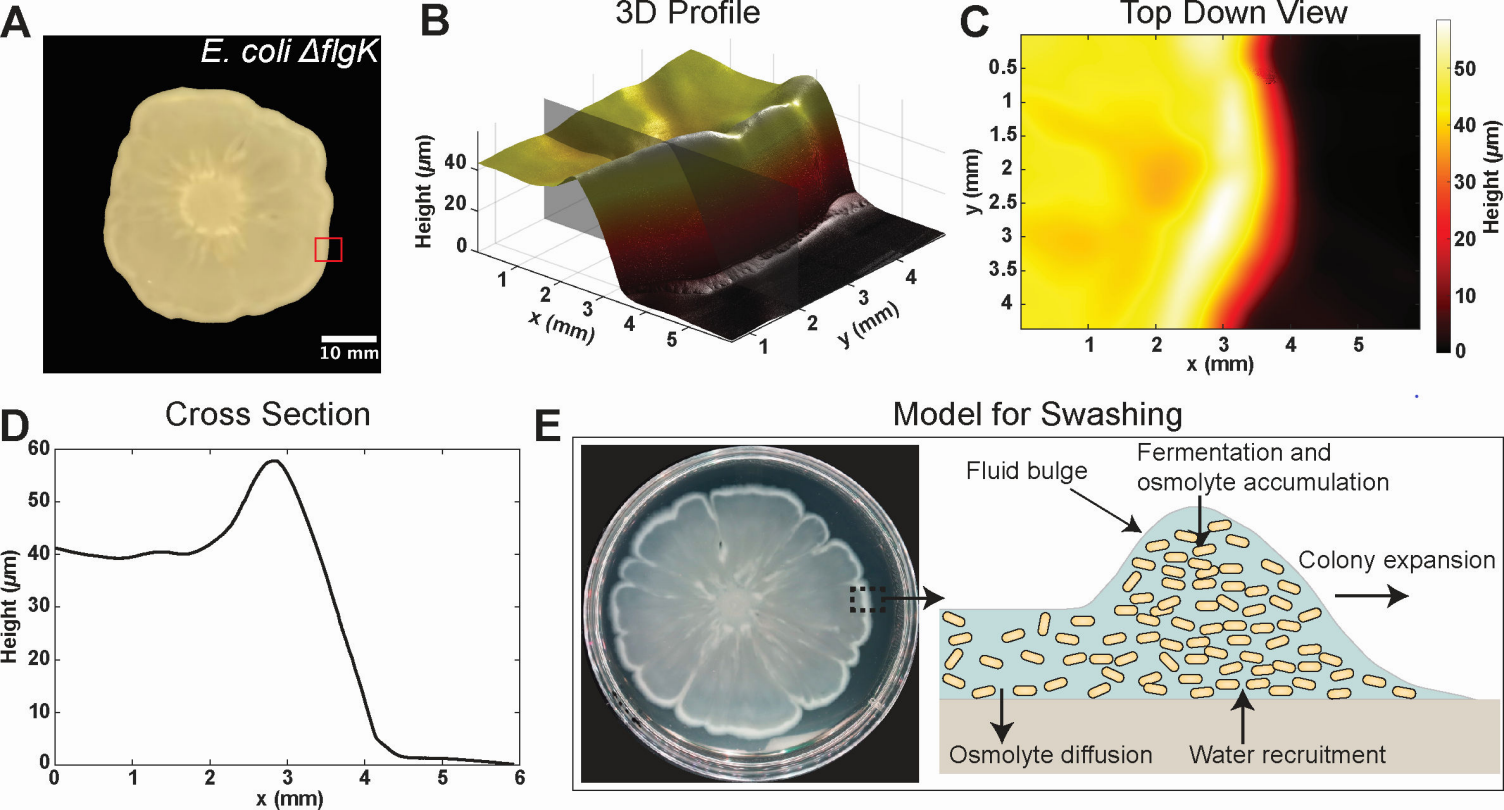
Colony topography and a model for bacterial swashing. (**A**) *E. coli* ∆*flgK* colony at 10 h post-inoculation. The red square marks the region analyzed by optical profilometry. (**B**) 3D height map of the colony’s leading edge. The height rises sharply from the agar surface (near zero) to ∼60 µm at the edge and then remains elevated at ∼40 µm across the interior. (**C**) Top-down view of the colony profile. The agar surface is to the right; the colony interior is to the left. (**D**) 2D cross-section of the colony, corresponding to the gray plane in (**B**). (**E**) Model for bacterial swashing. A bulge at the colony’s leading edge, consistent with optical profilometry data, contains a high density of cells. Fermentation of sugars by bacteria generates osmolytes, creating an osmotic gradient that draws water from the agar and drives colony expansion. Behind the front, the bulge diminishes as both osmolytes and water diffuse back into the agar. Colony image in (**E**) is duplicated from Fig. 1K.

Figure 4 presents the topography of a swashing *E. coli* ∆*flgK* colony. In Fig. 4A, the colony is imaged 10 h post-inoculation. The red box marks the region analyzed by optical profilometry, a non-contact method that maps surface height using light interference. The resulting 3D surface map (Fig. 4B) reveals a steep increase in height at the colony edge, peaking at around 60 µm, followed by a plateau of ∼40 µm in the interior. A top-down view (Fig. 4C) shows the agar surface on the right (0 µm) and the colony interior on the left. The fluid bulge at the leading edge—∼2 mm wide and ∼60 µm high—is clearly visible in the 2D cross-section (Fig. 4D). This fluid bulge grows with the colony, consistently remaining at the leading edge, suggesting it plays a key role in driving outward expansion.

## DISCUSSION

Our finding that *E. coli* and *Salmonella* mutants lacking functional flagella can still migrate rapidly on soft agar expands the known modes of surface migration in these species beyond those dependent on flagellar propulsion (2, 13–15). Our results show that, in fact, flagellar propulsion is dispensable for surface migration. We propose a model in which fermentation-driven osmotic flows drive colony expansion in filamentless *E. coli* and *Salmonella*. In view of this model, we term this process “swashing,” as it resembles a thin layer of water washing onto a beach, often carrying with it particulate and organic material. Swashing offers a new perspective on bacterial range expansion over wet porous surfaces such as agar, highlighting how otherwise non-motile cells can exploit the coupling between cellular metabolism and fluid physics to drive movement.

### Swashing is distinct from other mechanisms of propulsion-independent surface motility

Swashing exhibits unique characteristics that differentiate it from other mechanisms of propulsion-independent migration. A previous report described propulsion-independent surface migration in *Salmonella* on 0.3% agarose plates, dependent on the protein PagM (44). Swashing is different: it occurs on 0.55% agar, is faster, and does not require PagM, which is absent in the *Salmonella* strains (LT2 and derivatives) studied here. Swashing is also distinct from the “sliding” motility observed in several species (12). Sliding, driven by cell growth, is facilitated by surfactants (44, 54) that are not produced by the strains studied here. In contrast, swashing is inhibited by surfactants. In a colony that is expanding by a growth-based mechanism, the density of cells should remain high behind (i.e., inside) the advancing edge of the colony. This contrasts with what is seen in swashing colonies of the ∆*flgK* strain, where a thin zone of high cell density advances outward while leaving a zone of lower density behind (Fig. 4; Fig. S13). These distinctions highlight swashing as a distinct mechanism of range expansion, characterized by its independence from PagM, lack of surfactant facilitation, and distinctive cell density distribution during colony expansion.

### A model for swashing

The data collected in this study lead us to suggest the following descriptive model for swashing (Fig. 4E). At the colony edge, high cell density and elevated osmolarity—due to acetate and formate production—draw water from the agar. This fluid influx drives outward expansion and results in the formation of a bulge at the colony front. Behind the front, acetate accumulates and pH decreases, causing osmolyte production to slow. This asymmetry allows osmolytes to diffuse back into the agar, pulling fluid down and causing a ∼20 µm drop in height. In essence, fluid motion into the colony in the front and out of the colony behind the front generates a “wave” that cells ride outward. This model explains the role of glucose in promoting surface migration in *Salmonella* and *E. coli*. Beyond fueling growth, glucose fermentation creates osmotic flows that hydrate the leading edge and drive expansion.

### Could fermentation-generated osmotic gradients drive swashing?

The requirement for fermentable sugars (Fig. 3A) points to an association between swashing and fermentation. Furthermore, expansion is strongly correlated with media acidification (Fig. 3B), production of fermentation by-products (Fig. 3C), and is inhibited in the *ackA* knockout (Fig. 3D), all supporting fermentation’s critical role. In bacterial colonies growing on agar, oxygen concentration drops rapidly away from the colony-air and colony-agar interfaces due to consumption by cells near these boundaries. Experimental measurements (55) and computational models (56, 57) of colonies growing on 1.5%–2% agar both show that the colony interior becomes anoxic, promoting anaerobic metabolism. Our optical profilometry (Fig. 4) and line density scans (Fig. S13) further reveal that colony thickness as well as cell density are highest at the edge, both of which promote oxygen depletion. Even in well-oxygenated sections of the colony edge, cells with a surplus of glucose could be performing fermentation by overflow metabolism (58). Whether due to oxygen limitation or to overflow metabolism, swashing cells perform fermentation and generate fermentation by-products including acetate and formate (Fig. 3C). The acetate and formate concentrations, measured at ∼15 mM via plug biopsy (Fig. 3C), assume uniform distribution across the colony and agar, but production is localized to the colony. Assuming a colony height of ∼50 µm (Fig. 4D) and agar thickness of ∼4 mm, the actual osmolyte concentration in the colony can be much higher, in the molar range. Moreover, actual formate concentrations at the colony margin are likely to be higher than measured, as the spiked shape of the formate profile (Fig. 3C) suggests the re-acquisition of secreted formate. Nevertheless, the measured total acetate and formate (∼15 mM) represents a significant fraction of the osmolarity of LB-glucose swarm medium (around 250 mM) (59). Thus, the secreted acetate and formate likely create a strong osmotic gradient between the colony and the agar, driving fluid flow that could, in turn, enable swashing.

To further assess whether vertical osmotic influx could sustain colony spreading, we performed a simple order-of-magnitude estimate. As the colony is radially symmetric, we consider the “per unit circumference” forms of all relevant quantities. The bulge at the colony front is on the order of tens of microns higher than the interior (*h_b_* ∼ 20 µm) and about a millimeter wide (*w_b_* ∼ 1 mm). As the edge advances at speed *U* ∼ 10^−6^ m/s (1 µm/s), the volume (per unit circumference) that must be supplied per second is *h_b_U*. If water enters vertically through the agar beneath the bulge, the influx, *Jw_b_*, must balance the change in volume per second, which gives *J* ∼ *h_b_U*/*w_b_* ∼ 10^−8^ m/s. Here, *J* denotes the vertical Darcy flux per unit circumference. Applying Darcy’s law over an agar thickness *l* ∼ 10^−3^ m, water viscosity *µ* ∼ 10^−3^ Pa · s, and permeability *k* ∼ 10^−15^ m^2^ (60), this flux corresponds to an osmotic pressure gradient


$$\Delta \pi \;∼\;\frac{\mu lJ}{k}\;∼\;10\;Pa.$$

By van’t Hoff’s law, the corresponding vertical concentration difference is


$$\Delta C_{⊥}\;∼\;\frac{\Delta \pi }{RT}\;∼\;10^{-5}\;M,$$

where *R* is the gas constant and *T* is absolute temperature. Thus, according to these back-of-the-envelope estimates, the millimolar range osmolyte gradient estimated from the acetate and formate measurements is within the correct order of magnitude to sustain the necessary fluid influx.

### The role of capillary forces

Surfactants often aid surface expansion in bacteria, by affecting capillary force (surface tension), the tangential force acting at the fluid-air interface. Many bacteria, such as *P. aeruginosa* and *Bacillus subtilis*, produce surfactants to facilitate surface motility (33, 61, 62). However, in these cases, it is not surface tension itself but gradients in surface tension that drive expansion via Marangoni flows (63, 64). *Salmonella* and *E. coli* do not produce surfactants, suggesting that surface tension gradients are not involved in swashing.

The exogenous addition of Tween-80 (Fig. 2) lowers surface tension throughout the colony and, thus, has no effect on gradients. We speculate that Tween-80 inhibits swashing by lowering capillary forces that contribute to colony expansion, as suggested in scaling arguments by Srinivasan et al. (32). Indeed, our findings of reduced migration at lower surface tension are in agreement with the model predictions by Srinivasan et al. (32). However, unlike their findings of a power-law dependence on surface tension, we observed a sharp transition from expansion to no expansion with the concentration of Tween-80 (and thus surface tension) in the medium (Fig. 2). Additionally, the *B. subtilis* strain used by Srinivasan et al. (32) makes a surfactant, which would inhibit swashing. Overall, while surface tension (but not its gradient) is clearly a factor in swashing, its precise role remains to be elucidated.

### The role of flagellar components in swashing

Flagellar components, while not required for swashing, play a role in modulating it. The reduced migration of ∆*motA* cells is consistent with previous findings (15). It was surprising, however, that removing the filament restores outward migration in the ∆*motA* strain. This suggests that the failure of the ∆*motA* strain to migrate may stem from the presence of paralyzed filaments, rather than the absence of active propulsion. Non-rotating filaments could hinder movement by interacting with the surface or neighboring cells. This hindrance appears to be alleviated when ∆*motA* cells are mixed with ∆*flgK* cells, possibly by lowering the overall density of paralyzed filaments. These remain speculative interpretations, and other explanations are also possible and warrant further investigation.

How flagellar gene regulation influences, or is influenced by, swashing remains unclear. The absence of flagellar filament in the swashing strains could elevate FliA activity—responsible for regulating late-stage flagellar genes—by promoting constitutive export of FlgM, its antagonist. The lack of swashing in a ∆*fliA*∆*flgK* mutant appears to support this idea. However, a ∆*flgM*∆*flgK* mutant, which should have constitutively high FliA activity, also fails to swash, suggesting that FliA activity alone does not determine swashing ability. Nevertheless, these findings offer a useful starting point for future investigations into the role of gene regulation in swashing.

### Osmotic flow as a common thread in swashing and swarming

While swashing is clearly distinct from swarming, in its lack of dependence on flagella and its inhibition by surfactants, we speculate that the osmolyte-driven fluid flows may also contribute substantially to swarm expansion. Although such flows have long been recognized as important for swarming (31, 33, 34), their exact role has remained unclear. It has generally been supposed that they serve to create a fluid layer in which swarming cells move by flagellar propulsion. Our findings suggest that these flows might themselves drive colony expansion, even in the absence of flagellar activity.

Indeed, our model for swashing (Fig. 4) is very similar to the one proposed for swarming by Wu and Berg (27), but with a key distinction. Like us, Wu and Berg postulated that cellular metabolic activity leads to osmolyte accumulation near the colony front that draws water from the agar. However, they further suggested that flagellar rotation pumps fluid outward at the colony front, creating a water reservoir that the cells can swim into. Such pumping is not possible in our filamentless cells. In contrast, we propose that colony expansion, whether in swarming or in swashing, is driven by osmotically induced fluid influx and outflux. Like swashing colonies, WT swarming cells acidify the agar, indicating glucose fermentation (Fig. S14). The specific osmolyte(s) involved in swarming remain unidentified, but we suggest that acetate and formate are the osmolytes implicated in the swarming motility of *Salmonella* and *E. coli* (15).

Recent works offer additional support for these ideas. Bru et al. (33) recently questioned the role of flagellar propulsion in *P. aeruginosa* swarming, offering arguments that align with our findings. Our model also parallels that of Srinivasan et al. (32) for *B. subtilis* swarming, in which expansion is primarily driven by osmotic flux without explicit contribution from flagellar motility. Recent spatial transcriptomic data in *B. subtilis* show upregulation of fermentation genes at swarm front (65), consistent with a role for fermentation in generating osmotic gradients that hydrate the colony. However, unlike swashing, swarming in *P. aeruginosa* as well as *B. subtilis* is facilitated by surfactants, highlighting the subtleties involved in the underlying physical mechanisms.

In summary, we describe a mode of bacterial surface migration that does not rely on active propulsion and propose that it instead utilizes osmotic flows generated by the products of fermentative metabolism. Since key components of this mechanism—fermentation and semi-permeable wet surfaces—are widespread, we hypothesize that the physical drivers of swashing could also contribute to the range expansion of many bacterial species and other single-celled organisms. For instance, a similar and yet unexplained form of filamentless movement has also been observed in *Staphylococcus aureus* that might involve a similar process (66).

## Data Availability

All codes and data used in this manuscript are available from the GitHub repository https://github.com/wadhwalab/swashing.

## SUPPLEMENTAL MATERIAL

Supplemental figures and tables jb.00323-25-s0001.pdf Fig. S1 to S14; Tables S1 to S4.

ASM does not own the copyrights to Supplemental Material that may be linked to, or accessed through, an article. The authors have granted ASM a non-exclusive, world-wide license to publish the Supplemental Material files. Please contact the corresponding author directly for reuse.

## References

[1] Wadhwa N, Berg HC. 2022. Bacterial motility: machinery and mechanisms. Nat Rev Microbiol 20:161–173. doi:10.1038/s41579-021-00626-4 34548639

[2] Harshey RM. 2003. Bacterial motility on a surface: many ways to a common goal. Annu Rev Microbiol 57:249–273. doi:10.1146/annurev.micro.57.030502.091014 14527279

[3] Ottemann KM, Miller JF. 1997. Roles for motility in bacterial-host interactions. Mol Microbiol 24:1109–1117. doi:10.1046/j.1365-2958.1997.4281787.x 9218761

[4] Colin R, Ni B, Laganenka L, Sourjik V. 2021. Multiple functions of flagellar motility and chemotaxis in bacterial physiology. FEMS Microbiol Rev 45:fuab038. doi:10.1093/femsre/fuab038 34227665 PMC8632791

[5] Henrichsen J. 1972. Bacterial surface translocation: a survey and a classification. Bacteriol Rev 36:478–503. doi:10.1128/br.36.4.478-503.1972 4631369 PMC408329

[6] Berg HC, Anderson RA. 1973. Bacteria swim by rotating their flagellar filaments. Nature 245:380–382. doi:10.1038/245380a0 4593496

[7] Larsen SH, Adler J, Gargus JJ, Hogg RW. 1974. Chemomechanical coupling without ATP: the source of energy for motility and chemotaxis in bacteria. Proc Natl Acad Sci USA 71:1239–1243. doi:10.1073/pnas.71.4.1239 4598295 PMC388200

[8] Nakamura S, Minamino T. 2019. Flagella-driven motility of bacteria. Biomolecules 9:279. doi:10.3390/biom9070279 31337100 PMC6680979

[9] Hu H, Santiveri M, Wadhwa N, Berg HC, Erhardt M, Taylor NMI. 2022. Structural basis of torque generation in the bi-directional bacterial flagellar motor. Trends Biochem Sci 47:160–172. doi:10.1016/j.tibs.2021.06.005 34294545

[10] Craig L, Forest KT, Maier B. 2019. Type IV pili: dynamics, biophysics and functional consequences. Nat Rev Microbiol 17:429–440. doi:10.1038/s41579-019-0195-4 30988511

[11] McBride MJ. 2019. Bacteroidetes gliding motility and the type IX secretion system. Microbiol Spectr 7:10.1128/microbiolspec.psib-0002-2018. doi:10.1128/microbiolspec.psib-0002-2018 PMC11588200 30767845

[12] Hölscher T, Kovács ÁT. 2017. Sliding on the surface: bacterial spreading without an active motor. Environ Microbiol 19:2537–2545. doi:10.1111/1462-2920.13741 28370801

[13] Kearns DB. 2010. A field guide to bacterial swarming motility. Nat Rev Microbiol 8:634–644. doi:10.1038/nrmicro2405 20694026 PMC3135019

[14] Harshey RM. 1994. Bees aren’t the only ones: swarming in Gram‐negative bacteria. Mol Microbiol 13:389–394. doi:10.1111/j.1365-2958.1994.tb00433.x 7997156

[15] Harshey R M, Matsuyama T. 1994. Dimorphic transition in Escherichia coli and Salmonella typhimurium: surface-induced differentiation into hyperflagellate swarmer cells. Proc Natl Acad Sci USA 91:8631–8635. doi:10.1073/pnas.91.18.8631 8078935 PMC44660

[16] Smith DG. 1972. The Proteus swarming phenomenon. Sci Prog 60:487–506. 4601177

[17] Ulitzur S. 1974. Induction of swarming in Vibrio parahaemolyticus. Arch Microbiol 101:357–363. doi:10.1007/BF00455952 4455130

[18] Kearns DB, Losick R. 2003. Swarming motility in undomesticated Bacillus subtilis. Mol Microbiol 49:581–590. doi:10.1046/j.1365-2958.2003.03584.x 12864845

[19] Hernández F, Rodríguez E. 1993. The swarming phenomenon of Clostridium tetani. Rev Biol Trop 41:857–859. 7886258

[20] Wang Q, Frye JG, McClelland M, Harshey RM. 2004. Gene expression patterns during swarming in Salmonella typhimurium: genes specific to surface growth and putative new motility and pathogenicity genes. Mol Microbiol 52:169–187. doi:10.1111/j.1365-2958.2003.03977.x 15049819

[21] Deditius JA, Felgner S, Spöring I, Kühne C, Frahm M, Rohde M, Weiß S, Erhardt M. 2015. Characterization of novel factors involved in swimming and swarming motility in Salmonella enterica serovar Typhimurium. PLoS One 10:e0135351. doi:10.1371/journal.pone.0135351 26267246 PMC4534456

[22] Bhagwat AA, Young L, Smith AD, Bhagwat M. 2017. Transcriptomic analysis of the swarm motility phenotype of Salmonella enterica serovar Typhimurium mutant defective in periplasmic glucan synthesis. Curr Microbiol 74:1005–1014. doi:10.1007/s00284-017-1267-1 28593349

[23] Wozniak CE, Chevance FFV, Hughes KT. 2010. Multiple promoters contribute to swarming and the coordination of transcription with flagellar assembly in Salmonella. J Bacteriol 192:4752–4762. doi:10.1128/JB.00093-10 20639318 PMC2937404

[24] Kim W, Surette MG. 2004. Metabolic differentiation in actively swarming Salmonella. Mol Microbiol 54:702–714. doi:10.1111/j.1365-2958.2004.04295.x 15491361

[25] Dell’Arciprete D, Blow ML, Brown AT, Farrell FDC, Lintuvuori JS, McVey AF, Marenduzzo D, Poon WCK. 2018. A growing bacterial colony in two dimensions as an active nematic. Nat Commun 9:4190. doi:10.1038/s41467-018-06370-3 30305618 PMC6180060

[26] Li H, Shi X, Huang M, Chen X, Xiao M, Liu C, Chaté H, Zhang HP. 2019. Data-driven quantitative modeling of bacterial active nematics. Proc Natl Acad Sci USA 116:777–785. doi:10.1073/pnas.1812570116 30593562 PMC6338848

[27] Wu Y, Berg HC. 2012. Water reservoir maintained by cell growth fuels the spreading of a bacterial swarm. Proc Natl Acad Sci USA 109:4128–4133. doi:10.1073/pnas.1118238109 22371567 PMC3306679

[28] Mariconda S, Wang Q, Harshey RM. 2006. A mechanical role for the chemotaxis system in swarming motility. Mol Microbiol 60:1590–1602. doi:10.1111/j.1365-2958.2006.05208.x 16796690

[29] Chen BG, Turner L, Berg HC. 2007. The wetting agent required for swarming in Salmonella enterica serovar Typhimurium is not a surfactant. J Bacteriol 189:8750–8753. doi:10.1128/JB.01109-07 17905988 PMC2168935

[30] Be’er A, Ariel G. 2019. A statistical physics view of swarming bacteria. Mov Ecol 7:9. doi:10.1186/s40462-019-0153-9 30923619 PMC6419441

[31] Ping L, Wu Y, Hosu BG, Tang JX, Berg HC. 2014. Osmotic pressure in a bacterial swarm. Biophys J 107:871–878. doi:10.1016/j.bpj.2014.05.052 25140422 PMC4142250

[32] Srinivasan S, Kaplan CN, Mahadevan L. 2019. A multiphase theory for spreading microbial swarms and films. eLife 8. doi:10.7554/eLife.42697 PMC6491038 31038122

[33] Bru JL, Kasallis SJ, Zhuo Q, Høyland-Kroghsbo NM, Sep SA. 2023. Swarming of P. aeruginosa: through the lens of biophysics. Biophys Rev 4:031305. doi:10.1063/5.0128140 PMC10540860 37781002

[34] Yang A, Tang WS, Si T, Tang JX. 2017. Influence of physical effects on the swarming motility of Pseudomonas aeruginosa*.* Biophys J 112:1462–1471. doi:10.1016/j.bpj.2017.02.019 28402888 PMC5389960

[35] Kasallis S, Bru J-L, Chang R, Zhuo Q, Siryaporn A. 2023. Understanding how bacterial collectives organize on surfaces by tracking surfactant flow. Curr Opin Solid State Mater Sci 27:101080. doi:10.1016/j.cossms.2023.101080 37427092 PMC10327653

[36] Scriven LE, Sternling CV. 1960. The Marangoni effects. Nature 187:186–188. doi:10.1038/187186a0

[37] Giverso C, Verani M, Ciarletta P. 2016. Emerging morphologies in round bacterial colonies: comparing volumetric versus chemotactic expansion. Biomech Model Mechanobiol 15:643–661. doi:10.1007/s10237-015-0714-9 26296713

[38] Karlinsey JE. 2007. Lambda-Red genetic engineering in Salmonella enterica serovar Typhimurium. Methods Enzymol 421:199–209. doi:10.1016/S0076-6879(06)21016-4 17352924

[39] Mahan MJ, Slauch JM, Mekalanos JJ. 1993. Bacteriophage P22 transduction of integrated plasmids: single-step cloning of Salmonella typhimurium gene fusions. J Bacteriol 175:7086–7091. doi:10.1128/jb.175.21.7086-7091.1993 8226650 PMC206837

[40] Gibson DG, Young L, Chuang RY, Venter JC, Hutchison CA, Smith HO. 2009. Enzymatic assembly of DNA molecules up to several hundred kilobases. Nat Methods 6:343–345. doi:10.1038/nmeth.1318 19363495

[41] Ershov D, Phan M-S, Pylvänäinen JW, Rigaud SU, Le Blanc L, Charles-Orszag A, Conway JRW, Laine RF, Roy NH, Bonazzi D, Duménil G, Jacquemet G, Tinevez J-Y. 2022. TrackMate 7: integrating state-of-the-art segmentation algorithms into tracking pipelines. Nat Methods 19:829–832. doi:10.1038/s41592-022-01507-1 35654950

[42] Chevance FFV, Hughes KT. 2008. Coordinating assembly of a bacterial macromolecular machine. Nat Rev Microbiol 6:455–465. doi:10.1038/nrmicro1887 18483484 PMC5963726

[43] Murray TS, Kazmierczak BI. 2008. Pseudomonas aeruginosa exhibits sliding motility in the absence of type IV pili and flagella. J Bacteriol 190:2700–2708. doi:10.1128/JB.01620-07 18065549 PMC2293233

[44] Park S-Y, Pontes MH, Groisman EA. 2015. Flagella-independent surface motility in Salmonella enterica serovar Typhimurium. Proc Natl Acad Sci USA 112:1850–1855. doi:10.1073/pnas.1422938112 25624475 PMC4330729

[45] Niu C, Graves JD, Mokuolu FO, Gilbert SE, Gilbert ES. 2005. Enhanced swarming of bacteria on agar plates containing the surfactant Tween 80. J Microbiol Methods 62:129–132. doi:10.1016/j.mimet.2005.01.013 15823402

[46] Be’er A, Harshey RM. 2011. Collective motion of surfactant-producing bacteria imparts superdiffusivity to their upper surface. Biophys J 101:1017–1024. doi:10.1016/j.bpj.2011.07.019 21889437 PMC3164129

[47] Partridge JD, Harshey RM. 2013. Swarming: flexible roaming plans. J Bacteriol 195:909–918. doi:10.1128/JB.02063-12 23264580 PMC3571328

[48] Warrell DL, Zarrella TM, Machalek C, Khare A. 2024. Interspecies surfactants serve as public goods enabling surface motility in Pseudomonas aeruginosa. J Bacteriol 206:e0028124. doi:10.1128/jb.00281-24 39235232 PMC11500613

[49] Inoue T, Shingaki R, Hirose S, Waki K, Mori H, Fukui K. 2007. Genome-wide screening of genes required for swarming motility in Escherichia coli K-12. J Bacteriol 189:950–957. doi:10.1128/JB.01294-06 17122336 PMC1797309

[50] Encheva V, Shah HN, Gharbia SE. 2009. Proteomic analysis of the adaptive response of Salmonella enterica serovar Typhimurium to growth under anaerobic conditions. Microbiology (Reading) 155:2429–2441. doi:10.1099/mic.0.026138-0 19389776

[51] Bettenbrock K, et al.. 2014. Towards a systems level understanding of the oxygen response of *Escherichia coli*, p 65–114. In Poole RK (ed), Advances in Microbial Physiology. Academic Press. 10.1016/B978-0-12-800143-1.00002-6 24797925

[52] Garcia-Gutierrez E, Chidlaw AC, Le Gall G, Bowden SD, Tedin K, Kelly DJ, Thompson A. 2016. A comparison of the ATP generating pathways used by S. typhimurium to fuel replication within human and murine macrophage and epithelial cell lines. PLoS One 11:e0150687. doi:10.1371/journal.pone.0150687 26930214 PMC4773185

[53] Rossmann R, Sawers G, Böck A. 1991. Mechanism of regulation of the formate-hydrogenlyase pathway by oxygen, nitrate, and pH: definition of the formate regulon. Mol Microbiol 5:2807–2814. doi:10.1111/j.1365-2958.1991.tb01989.x 1779767

[54] Brown II, Häse CC. 2001. Flagellum-independent surface migration of Vibrio cholerae and Escherichia coli. J Bacteriol 183:3784–3790. doi:10.1128/JB.183.12.3784-3790.2001 11371543 PMC95256

[55] Peters AC, Wimpenny JWT, Coombs JP. 1987. Oxygen profiles in, and in the agar beneath, colonies of Bacillus cereus, Staphylococcus albus and Escherichia coli. Microbiology (Reading, Engl) 133:1257–1263. doi:10.1099/00221287-133-5-1257 3116170

[56] Pirt SJ. 1967. A kinetic study of the mode of growth of surface colonies of bacteria and fungi. J Gen Microbiol 47:181–197. doi:10.1099/00221287-47-2-181 6045659

[57] Kannan H, Sun P, Çağlar T, Yao P, Taylor BR, Sahu K, Ge D, Mori M, Warren M, Kleinfeld D, Dong J, Li B, Hwa T. 2023. Spatiotemporal development of growth and death zones in expanding bacterial colonies driven by emergent nutrient dynamics. Microbiology. doi:10.1101/2023.08.27.554977 PMC12106844 40419492

[58] Basan M, Hui S, Okano H, Zhang Z, Shen Y, Williamson JR, Hwa T. 2015. Overflow metabolism in Escherichia coli results from efficient proteome allocation. Nature 528:99–104. doi:10.1038/nature15765 26632588 PMC4843128

[59] Rothe M, Alpert C, Engst W, Musiol S, Loh G, Blaut M. 2012. Impact of nutritional factors on the proteome of intestinal Escherichia coli: induction of OxyR-dependent proteins AhpF and Dps by a lactose-rich diet. Appl Environ Microbiol 78:3580–3591. doi:10.1128/AEM.00244-12 22427493 PMC3346377

[60] Araujo G, Chen W, Mani S, Tang JX. 2019. Orbiting of flagellated bacteria within a thin fluid film around micrometer-sized particles. Biophys J 117:346–354. doi:10.1016/j.bpj.2019.06.005 31248602 PMC6700674

[61] Caiazza NC, Shanks RMQ, O’Toole GA. 2005. Rhamnolipids modulate swarming motility patterns of Pseudomonas aeruginosa. J Bacteriol 187:7351–7361. doi:10.1128/JB.187.21.7351-7361.2005 16237018 PMC1273001

[62] Kinsinger RF, Shirk MC, Fall R. 2003. Rapid surface motility in Bacillus subtilis is dependent on extracellular surfactin and potassium ion. J Bacteriol 185:5627–5631. doi:10.1128/JB.185.18.5627-5631.2003 12949115 PMC193742

[63] Fauvart M, Phillips P, Bachaspatimayum D, Verstraeten N, Fransaer J, Michiels J, Vermant J. 2012. Surface tension gradient control of bacterial swarming in colonies of Pseudomonas aeruginosa. Soft Matter 8:70–76. doi:10.1039/C1SM06002C

[64] Trinschek S, John K, Thiele U. 2018. Modelling of surfactant-driven front instabilities in spreading bacterial colonies. Soft Matter 14:4464–4476. doi:10.1039/C8SM00422F 29796452

[65] Jeckel H, Nosho K, Neuhaus K, Hastewell AD, Skinner DJ, Saha D, Netter N, Paczia N, Dunkel J, Drescher K. 2023. Simultaneous spatiotemporal transcriptomics and microscopy of Bacillus subtilis swarm development reveal cooperation across generations. Nat Microbiol 8:2378–2391. doi:10.1038/s41564-023-01518-4 37973866 PMC10686836

[66] Davies MC, Pollitt EJG. 2024. Staphylococcus aureus gliding comets: formation and observation. bioRxiv. doi:10.1101/2024.12.17.628996

