# Supplementary material for: Swashing: a propulsion-independent form of bacterial surface migration
Source: J Bacteriol. 2025 Nov 3;207(11):e00323-25. doi: 10.1128/jb.00323-25 (PMC12632270; doi:10.1128/jb.00323-25)
Supplement: Supplemental figures and tables — Fig. S1 to S14; Tables S1 to S4. [file jb.00323-25-s0001.pdf]

Supplemental Information for

Swashing: A propulsion-independent form of bacterial surface migration

Justin Panich *et al.*

This PDF file includes:

Figs. S1 to S14
Tables S1 to S4
Reference (1-2)

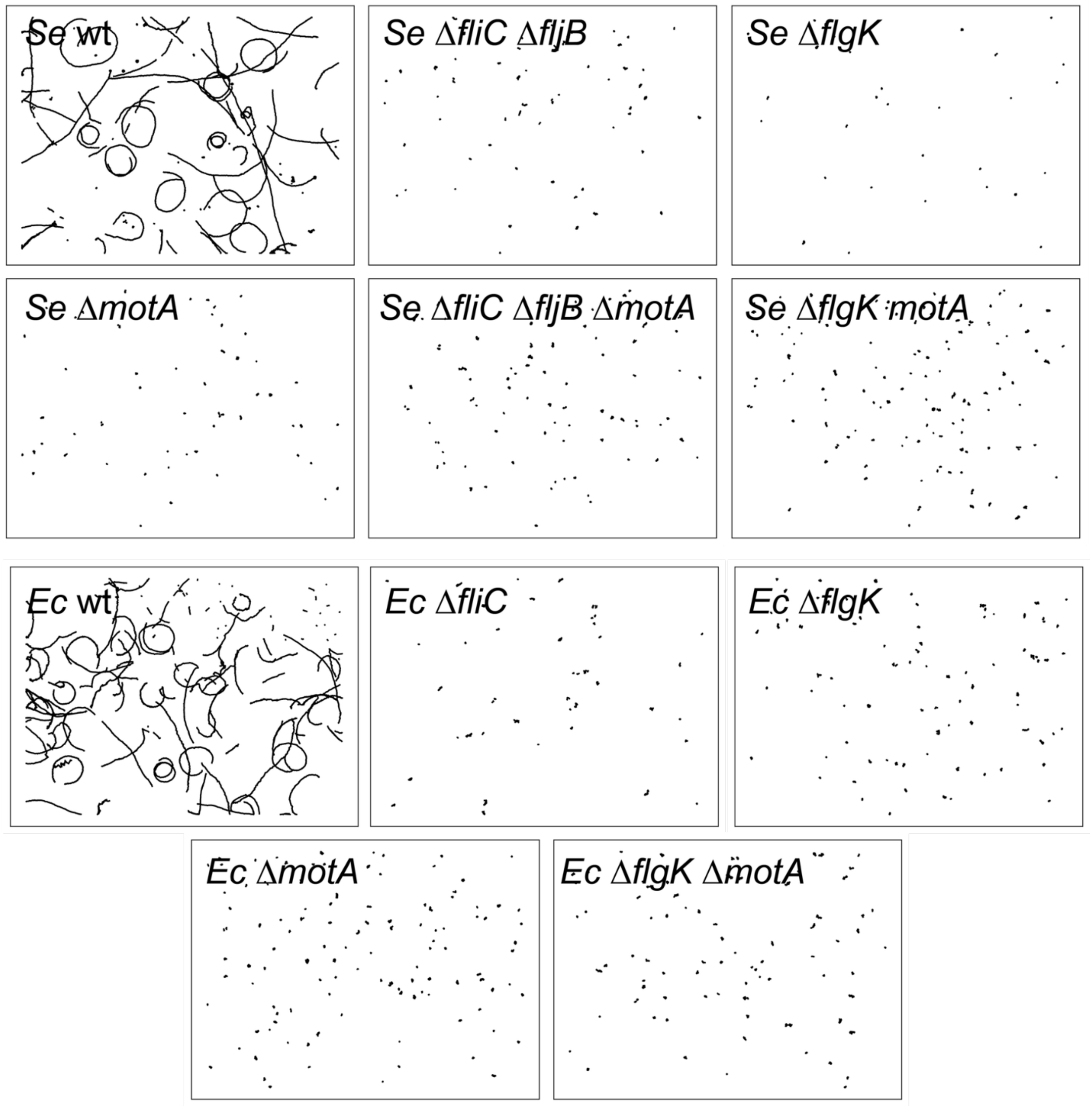

Figure S1. ***Salmonella* and *E. coli* microscopy and cell tracking.**

Salmonella (first six panels): WT cells swim using run and tumble motion. In contrast, cells lacking either the filament (Δ*fliC* Δ*fljB;* Δ*flgK;* Δ*flgL*) or the stator proteins (Δ*motA;* Δ*motB*) do not swim.

E. coli (last five panels): WT cells swim using run and tumble motion. In contrast, cells lacking either the filament (Δ*fliC;* Δ*flgK*) or the stator proteins (Δ*motA;* Δ*motB*) or both (Δ*motA*Δ*flgK*) are unable to swim.

The swimming patterns seen for wt cells are consistent with clockwise circular motion reported for cells interacting with the boundary, in this case, the glass coverslip [1].

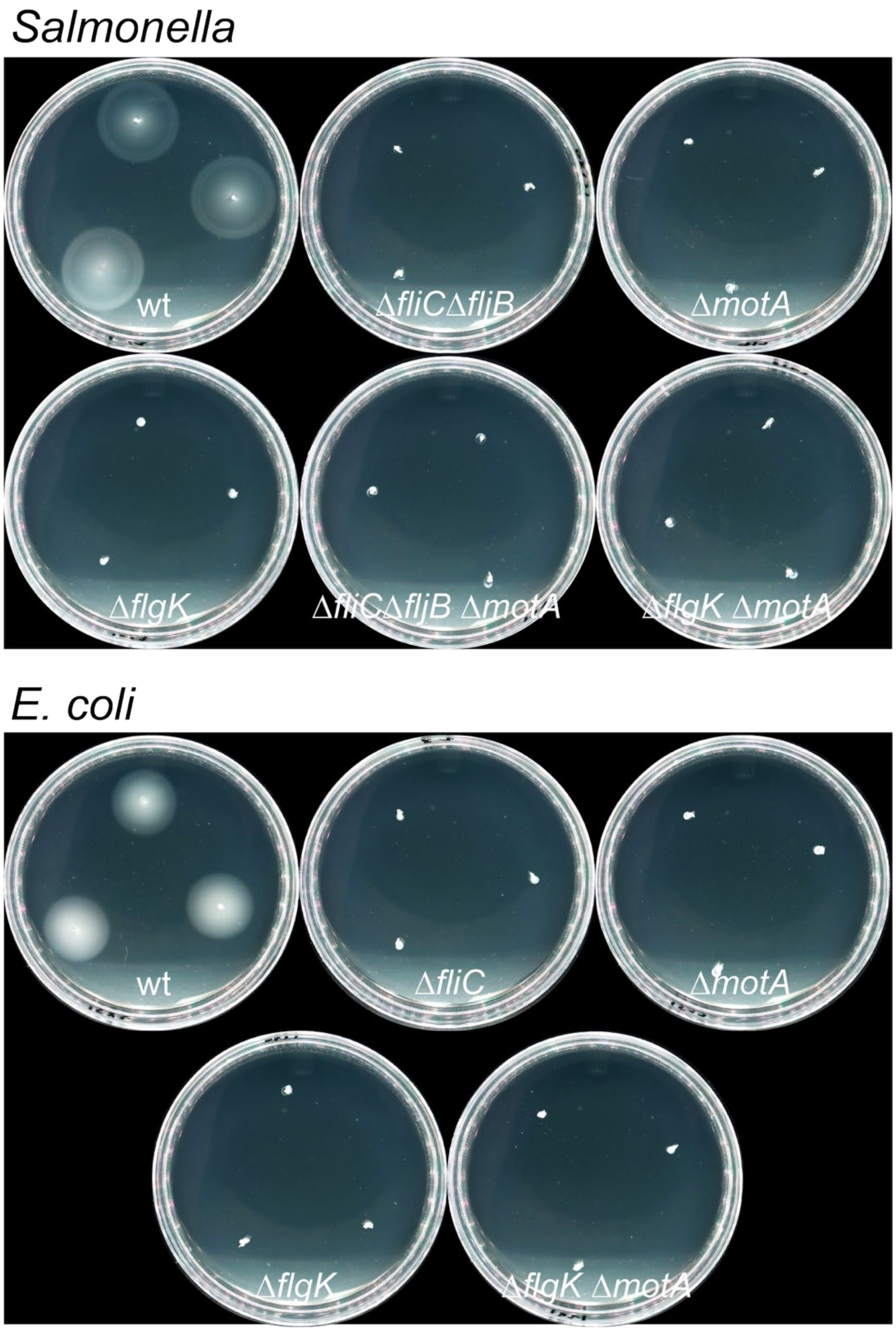

Figure S2. ***Salmonella*** **and** ***E. coli*** **motility assays in 0.30% agar swim plates.**

Salmonella (first six plates): WT cells migrate outwards, showing swimming behavior. In contrast, cells lacking either the filament (Δ*fliC* Δ*fljB;* Δ*flgK;* Δ*flgL*) or the stator proteins (Δ*motA*) are unable to migrate outwards.

E. coli (last five plates): WT cells migrate outwards, showing swimming behavior. In contrast, cells lacking either the filament (Δ*fliC;* Δ*flgK*), the stator proteins (Δ*motA*), or both (Δ*motA*Δ*flgK*) are unable to migrate outwards.

Each plate shows three biological replicates.

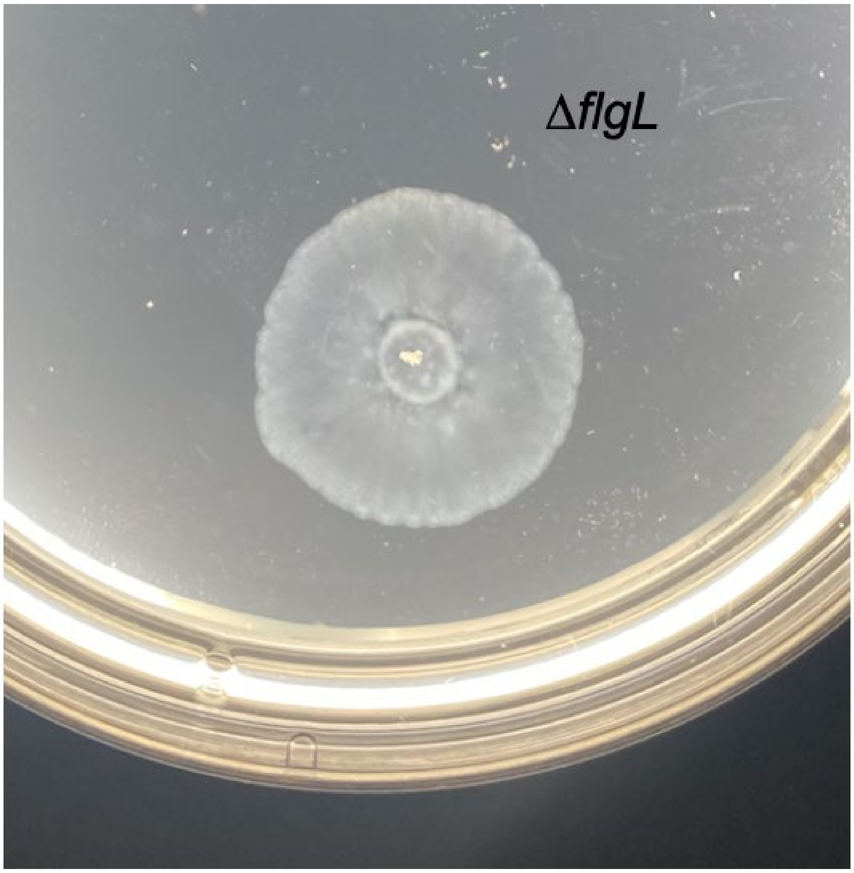

Figure S3. Migration of *Salmonella* Δ*flgL*.
Migration of *Salmonella* Δ*flgL* cells on a swarm plate, imaged at 8 hours.

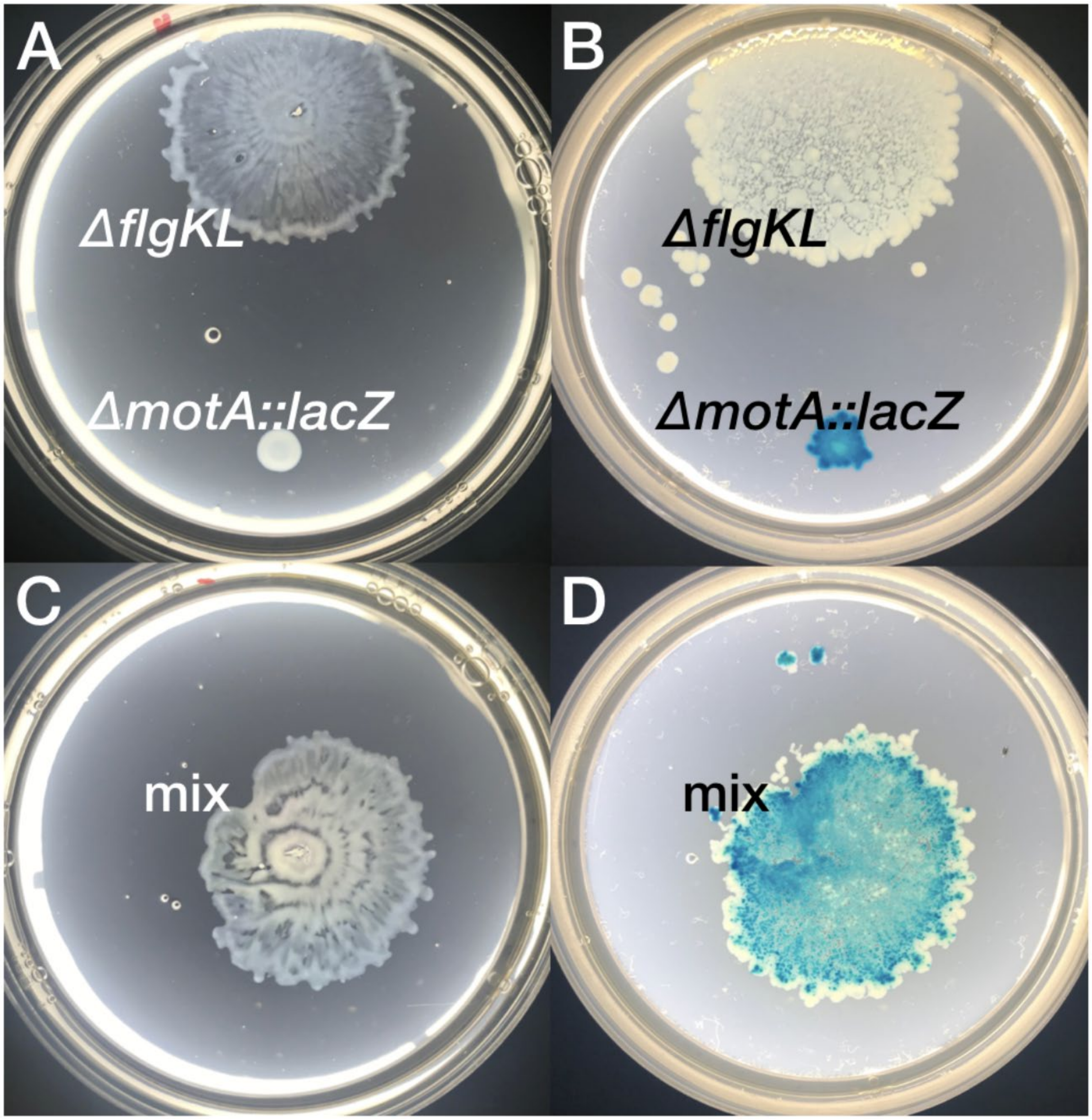

Figure S4. Migration of co-inoculated ∆*motA* and ∆*flgKL Salmonella* strains.

Salmonella ∆*motA* shows diminished migration relative to ∆*flgKL* (A), whereas a 1:1 mixture of ∆*motA* with ∆*flgKL* demonstrates uninhibited expansion (C). On X-galactose plates, a lacZ knock-in of ∆*motA* appears blue (B) and migrates to the edge of the swashing mixture alongside ∆*flgKL* (D).

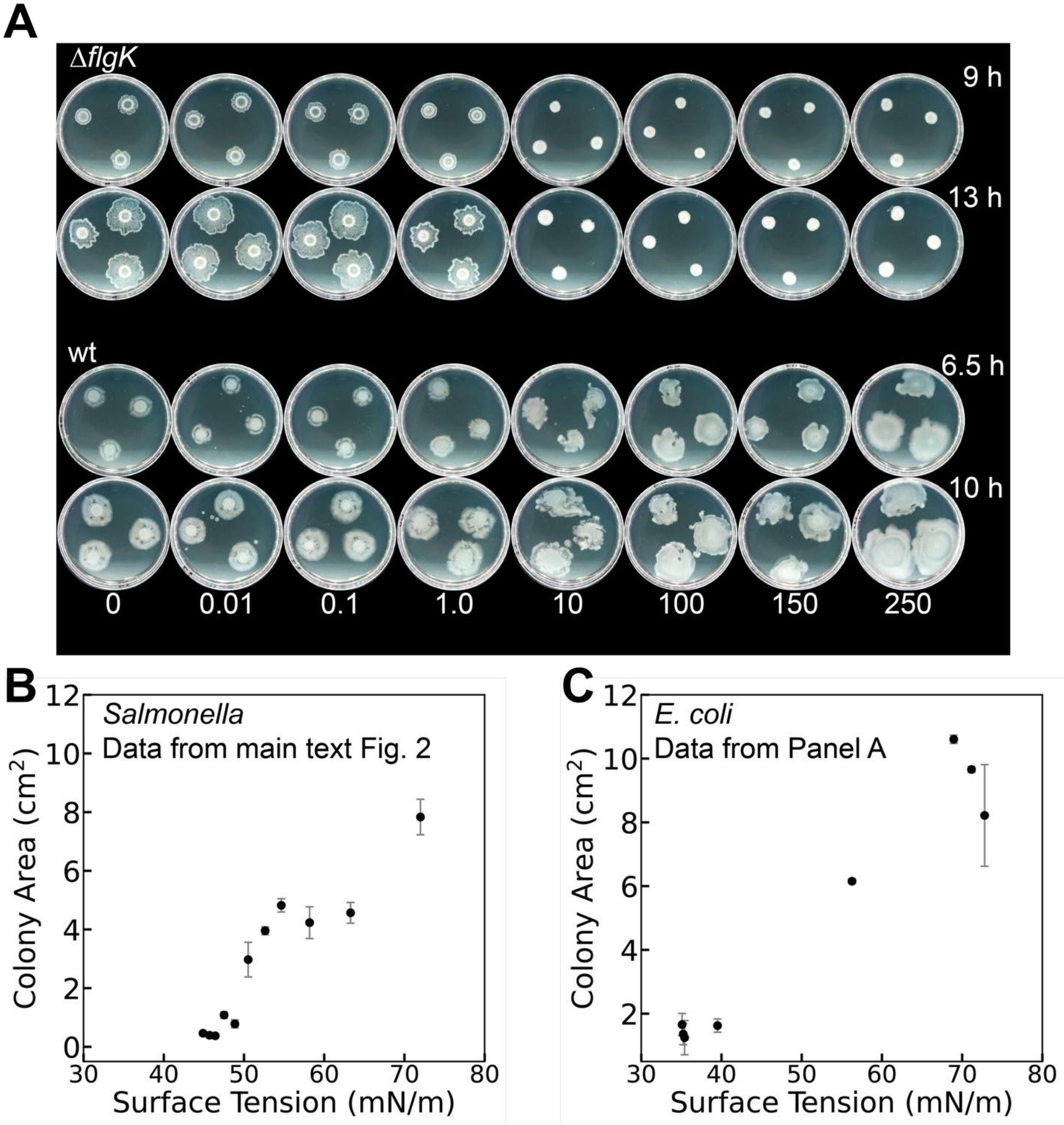

Figure S5. Inhibition of *E. coli* Δ*flgK* and enhancement of swarming by surfactant, and quantification of surfactant results. (A) Inhibition of *E. coli* Δ*flgK* swashing, and enhancement of swarming, by surfactant, Tween-80, and quantification of colony radii of *Salmonella* Δ*flgK* (B) and *E. coli* Δ*flgK* (C) for the various Tween-80 concentrations tested. Data in B correspond to main text Fig. 2. Surface tension values for the Tween-80 concentrations tested are estimated based on data from Pogorzelski et al. [2].

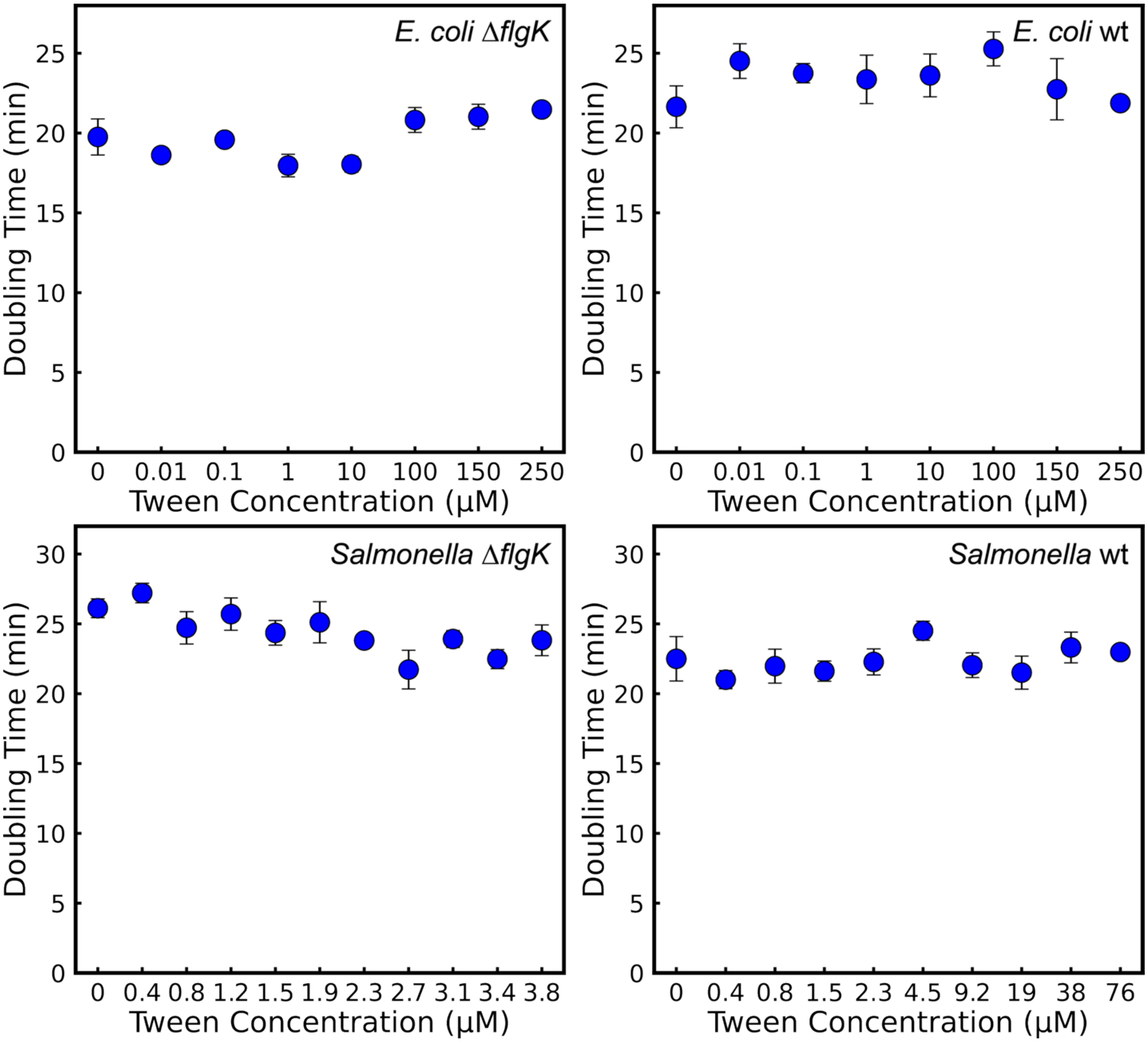

Figure S6. Tween-80 does not affect *Salmonella* or *E. coli* growth.

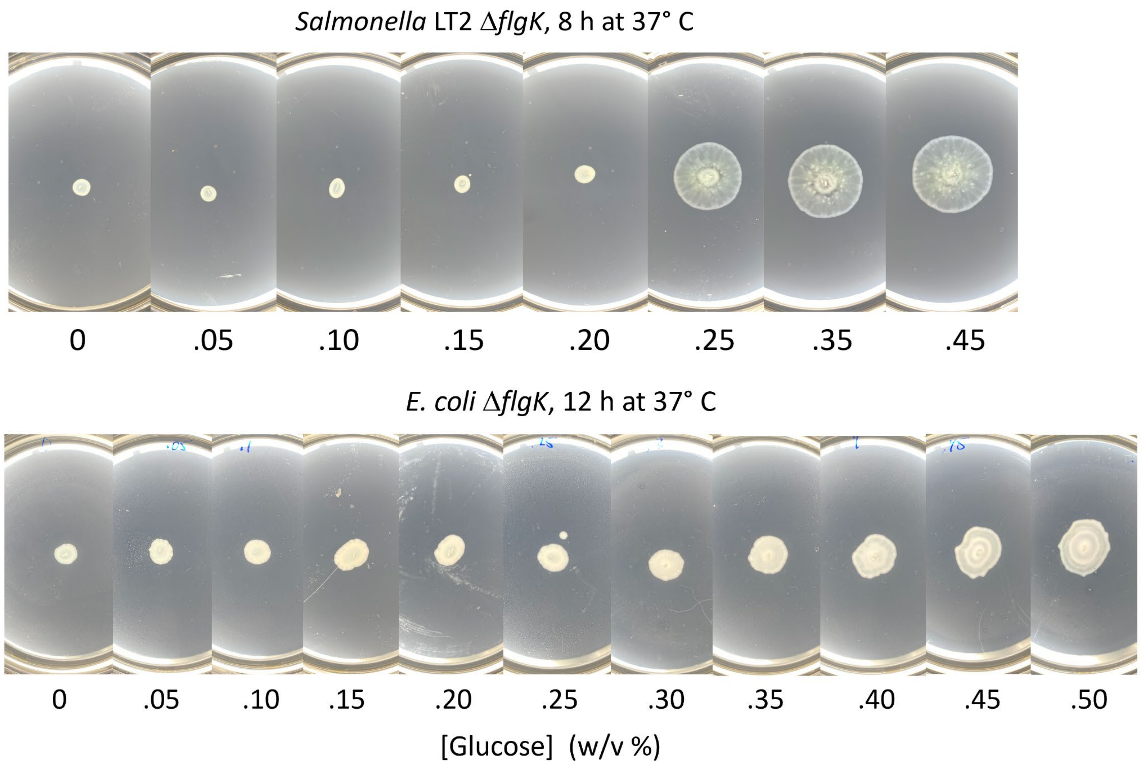

Figure S7. Migration of *Salmonella* Δ*flgK* and *E. coli* Δ*flgK* both depend on glucose. Surface migration of both *Salmonella* and *E. coli* Δ*flgK* strains ceased at glucose concentrations below 0.3%.

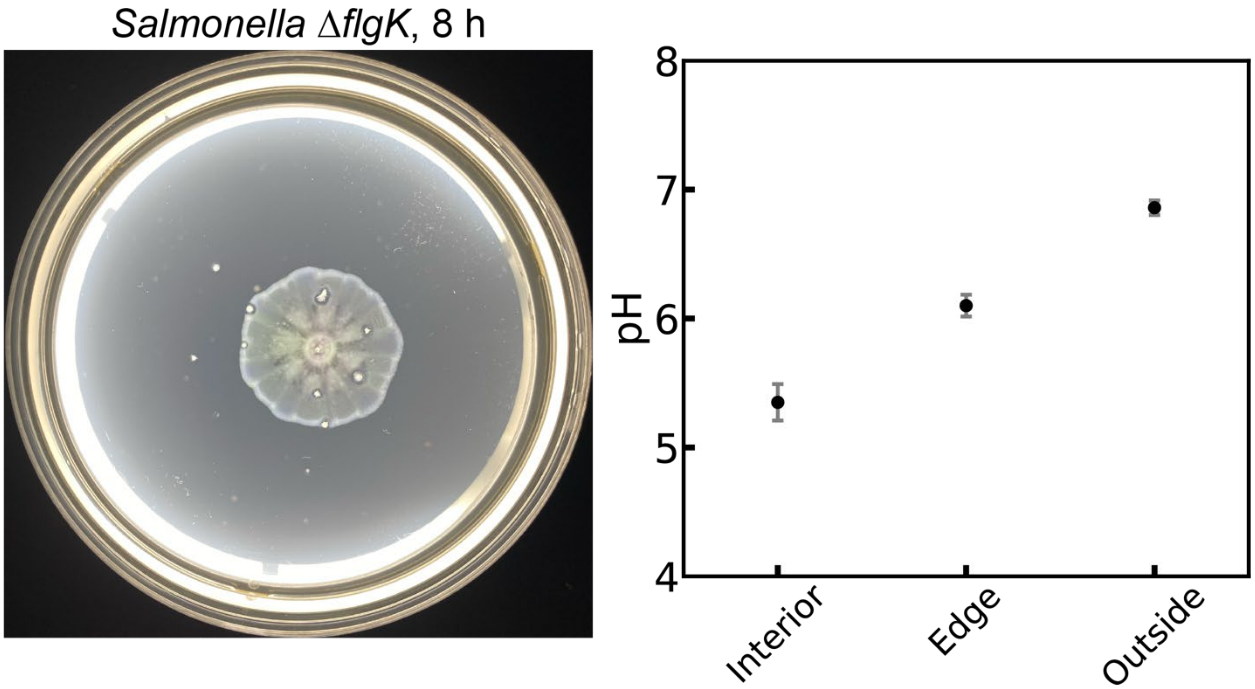

Figure S8. Quantification of pH inside, at the edge, and outside a swashing Δ*flgK Salmonella* colony.
pH measurements were obtained with an electrode for Δ*flgK Salmonella* swashing colonies at 8 h post-inoculation. The electrode left visible marks (seen as white dots) on the expanding colony. pH decreases at the edge of the colony, relative to the fresh agar outside, and further decreases towards the colony interior. Data shown as averages of eight measurements across two biological replicates, with standard deviation plotted as error bars.

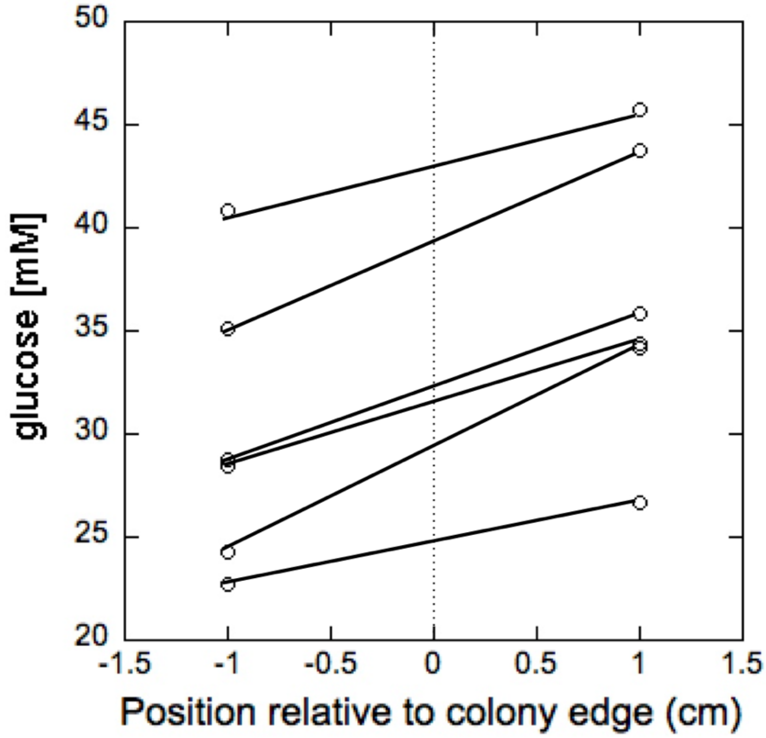

Figure S9. Measurements of glucose in front and behind the colony edge for expanding Δ*flgK* *Salmonella*.

Glucose concentration decreased by an average of 7 mM across the colony edge. Concentrations were obtained through the same procedure as in Fig. 3C.

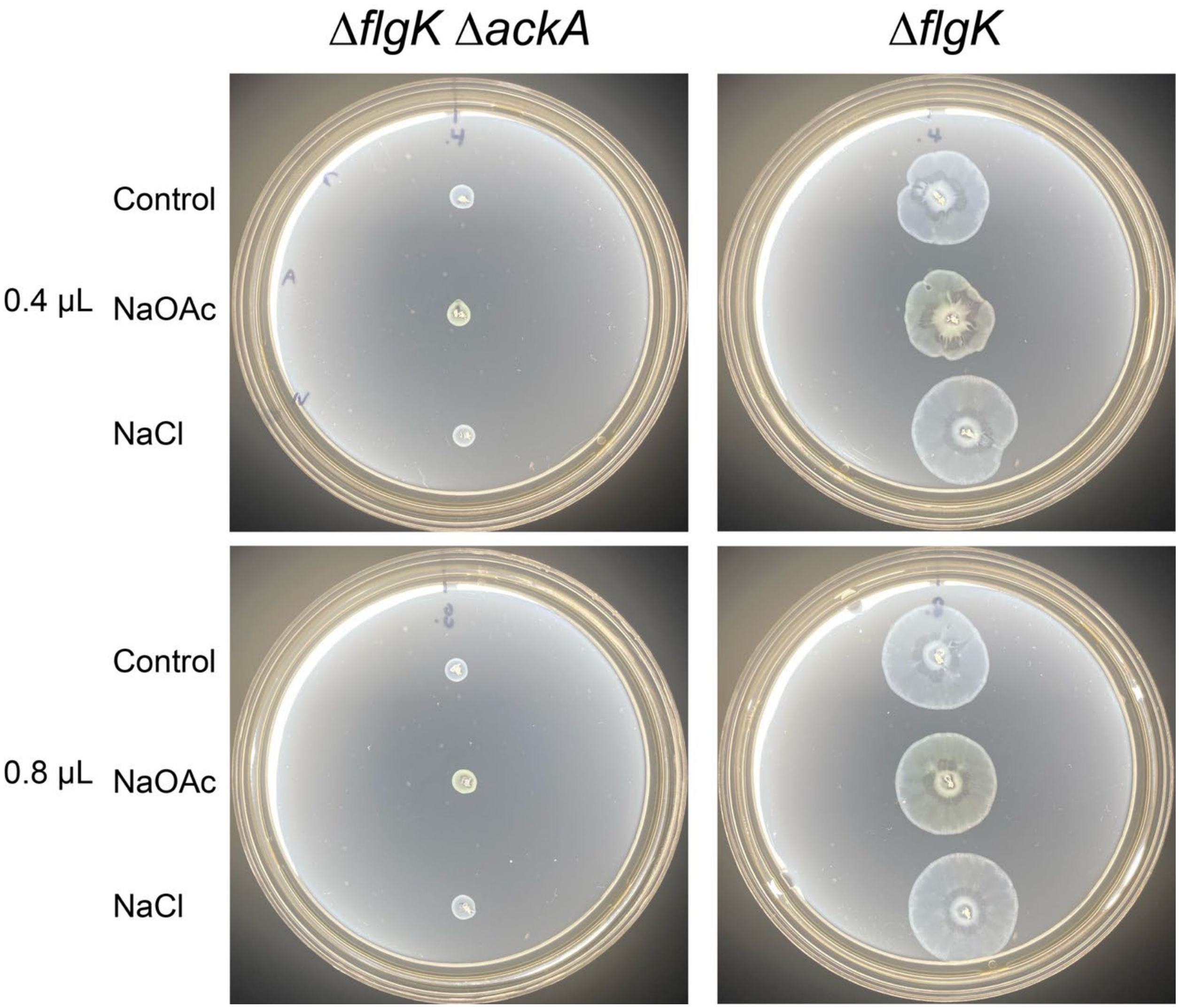

Figure S10. Exogenous acetate addition to the inoculation spot of a Δ*flgK*Δ*ackA* *Salmonella* strain did not restore swashing expansion.

Salmonella Δ*flgK*Δ*ackA* and Δ*flgK* mutants were inoculated into plates. To the initial 4 µL inoculant, either 0.4 µL or 0.8 µL of water (control), 1 M Na acetate (pH 6.9), or 1 M NaCl was added.

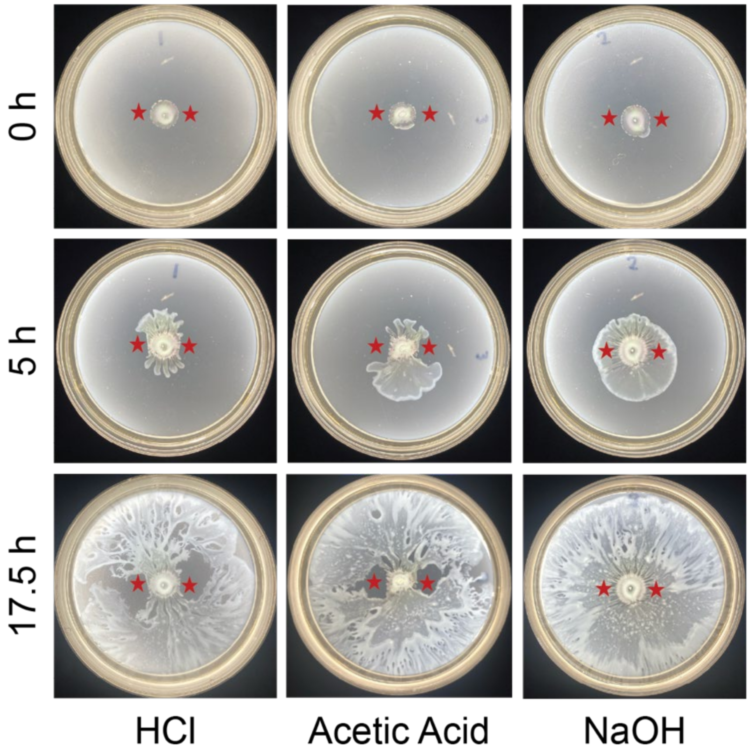

Figure S11. Acid spotting inhibits swashing *Salmonella*, whereas base slightly increases it.
4 µL of ∆*flgK Salmonella* were inoculated into the center of plates, spotted with either 1M hydrochloric acid, sodium hydroxide, or acetic acid, and were imaged at 0, 5, and 17.5 hours of incubation at 37 °C. The red stars indicate the locations of the acid or base drops.

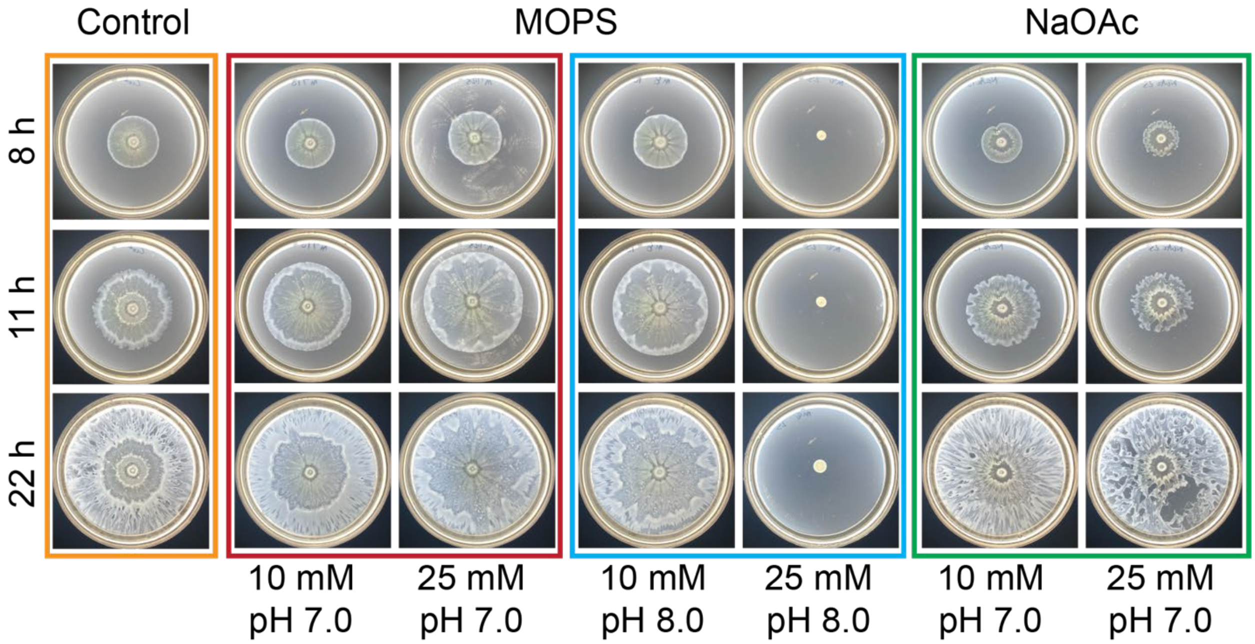

Figure S12. Buffering changes the rate and morphology of swashing *Salmonella*.

4 µL of Δ*flgK Salmonella* were inoculated into the center of plates and were imaged at 8, 11, and 22 hours of incubation at 37 °C. The agar plates contained either MOPS (a buffer, outlined in red at pH 7.0 and blue at pH 8.0), sodium acetate (a buffer and product of fermentation, outlined in green), or control (outlined in orange).

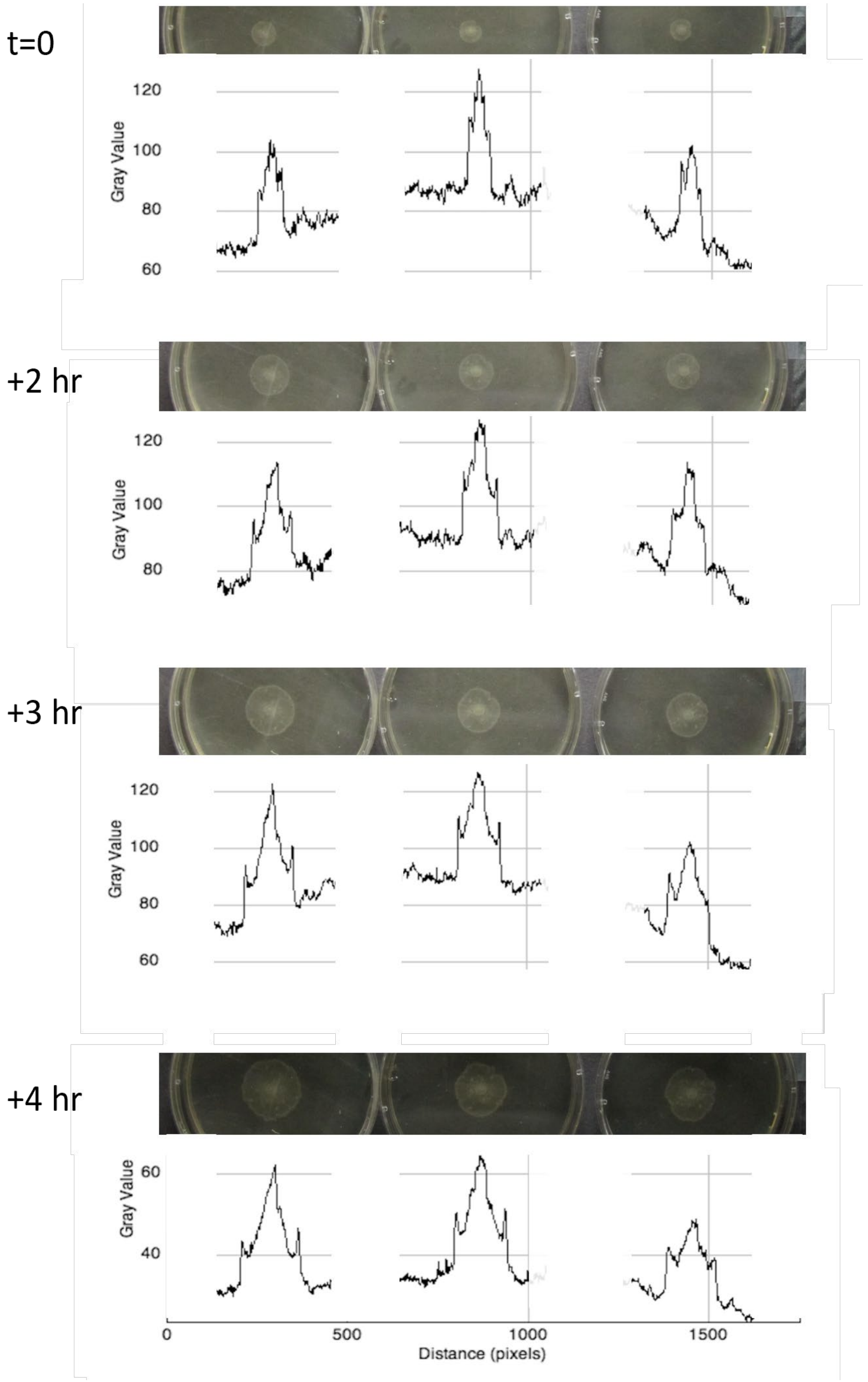

Figure S13. Densitometry measurements of a swashing ∆*flgK Salmonella* colony.

Densitometry measurements at the margin of swashing Δ*flgK Salmonella* colonies. Each row represents 3 biological replicates, and four different time points are shown.

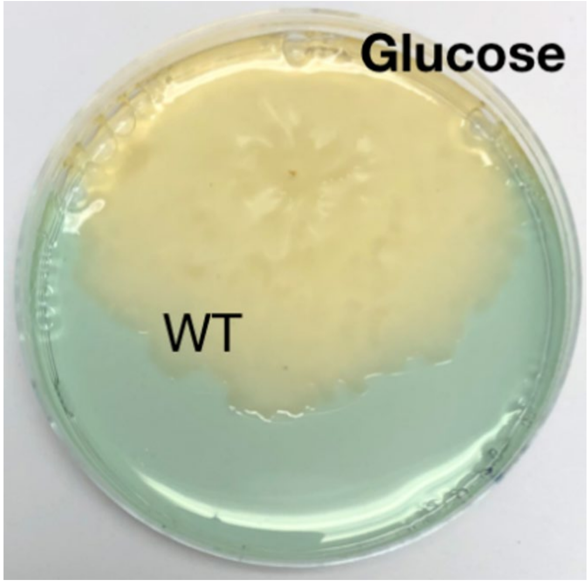

Figure S14. Fermentation of glucose by WT *Salmonella* growing on swarm plates.

To assess the pH of wild-type *Salmonella* growing on swarm plates, we used bromothymol blue, a pH indicator that turns yellow below pH 6.0 and blue above pH 7.6. In the presence of 0.5% (w/v) glucose, the medium acidified, indicating glucose fermentation by the bacteria.

Mutation	Δ*flgK*	Δ*flgL*	Δ*flgKL*
Δ*fliA*	-	-	-
Δ*flgM*	-	-	-

Table S1. Swashing behavior of *Salmonella* Δ*fliA and* Δ*flgM* hook-filament-connector double knockouts.

Double-knockout *Salmonella* colonies were incubated at 37° C. In the table, a "–" indicates a colony diameter less than 15 mm when plates were imaged at 12 h. A 15 mm colony diameter represents a small fraction, approximately 15%, of the diameter of the agar plate, and therefore represents a confined, non-expanding colony.

Strain Name	Background	Genotype	Source
JP1	*S. enterica* LT2	w.t.	K. Hughes
JP719	*S. enterica* LT2	Δ*flgK2408*	K. Hughes
JP720	*S. enterica* LT2	Δ*flgL2403*	K. Hughes
JP799	*S. enterica* LT2	Δ*fliC* Δ*fljB*	K. Hughes
JP1049	*S. enterica* LT2	Δ*motA::tetRA* Δ*fliC* Δ*fljB*	This study
JP1057	*S. enterica* LT2	Δ*motA::tetRA*	This study
JP1058	*S. enterica* LT2	Δ*motA::tetRA* Δ*flgK2408*	This study
JP1117	*S. enterica* LT2	Δ*flgKL*	K. Hughes
TH29200	*S. enterica* LT2	Δ*flgK ackA::mudJ*	This study
DBS710	*S. enterica* LT2	Δ*flgK srfB::mudJ*	This study
JP1533	*S. enterica* LT2	Δ*motA::lacZ*	This study

Table S2. *Salmonella* strains used in this study.

Strain Name	Background	Genotype	Source
JP1586	*E. coli K12 MG1655 F- lambda- mot+*	w.t.	K. Hughes
JP1593	*E. coli K12 MG1655 F- lambda- mot+*	Δ*motA::tetRA*	This study
JP1594	*E. coli K12 MG1655 F- lambda- mot+*	Δ*flgK::tetRA*	This study
JP1621	*E. coli K12 MG1655 F- lambda- mot+*	Δ*flgK* Δ*motA::tetRA*	This study
DFB28	*Escherichia coli* RP437	Δ*fliC*	This study

Table S3. *E. coli* strains used in this study.

Plasmid Name (resistance)	Clone	Source
pKD46 (ApTs)	Lambda red proteins	D. Blair
pMS421 (Sp)	vector	K. Hughes
pJP6 (Sp)	pMS421::*fljA*	This study

Table S4. **Plasmids used in this study.**

REFERENCES

1. Lauga, E., DiLuzio, W.R., Whitesides, G.M., and Stone, H.A. (2006). Swimming in circles: motion of bacteria near solid boundaries. Biophys J *90*, 400–412. https://doi.org/10.1529/biophysj.105.069401.

2. Pogorzelski, S., Watrobska-Swietlikowska, D., and Sznitowska, M. (2012). Surface tensometry studies on formulations of surfactants with preservatives as a tool for antimicrobial drug protection characterization. Journal of Biophysical Chemistry 3, 324–333. https://doi.org/10.4236/jbpc.2012.34040.
